# Supplementary material for: IRX3 controls a SUMOylation-dependent differentiation switch in adipocyte precursor cells
Source: Nat Commun. 2025 Aug 6;16:7248. doi: 10.1038/s41467-025-62361-1 (PMC12328774; doi:10.1038/s41467-025-62361-1)
Supplement: Supplementary file 1 — Supplementary Information [file 41467_2025_62361_MOESM1_ESM.pdf]

# Supplementary Information

## IRX3 controls a SUMOylation-dependent differentiation switch in adipocyte precursor cells

Jan-Inge Bjune[1,2,3], Samantha Laber[4], Laurence Lawrence-Archer[1,3], Patrizia M.C. Nothnagel[5], Shuntaro Yamada[6], Xu Zhao[5], Pouda Panahandeh Strømland[3], Niyaz Al-Sharabi[6], Kamal Mustafa[6], Pål R. Njølstad[1,7], Melina Claussnitzer[1,8,9,10], Roger D. Cox[4], Pierre Chymkowitch[5*], Gunnar Mellgren[1,2,3*†] and Simon N. Dankel[1,2,3*†]

[1] Mohn Research Center for Diabetes Precision Medicine, Department of Clinical Science, University of Bergen, N-5021 Bergen, Norway

[2] Mohn Nutrition Research Laboratory, Department of Clinical Science, University of Bergen, N-5021 Bergen, Norway

[3] Hormone Laboratory, Department of Medical Biochemistry and Pharmacology, Haukeland University Hospital, N-5021 Bergen, Norway

[4] Medical Research Council Harwell Institute, Mammalian Genetics Unit, Harwell Campus, Oxfordshire, OX11 0RD, UK

[5] Department of Biosciences, Faculty of Mathematics and Natural Sciences, University of Oslo, P.O. Box 1066 Blindern, 0316 Oslo, Norway

[6] Center of Translational Oral Research-Tissue engineering, Department of Clinical Dentistry, University of Bergen, N-5021 Bergen, Norway

[7] Department of Pediatrics and Adolescents, Haukeland University Hospital, N-5021 Bergen, Norway

[8] The Novo Nordisk Foundation Center for Genomic Mechanisms of Disease, Broad Institute of MIT and Harvard, Cambridge, MA 02142, USA

[9] Diabetes Unit and Center for Genomic Medicine, Massachusetts General Hospital, Boston,

Massachusetts, 02114, USA

[10] Department of Medicine, Harvard Medical School, Boston, MA

[†]Contributed equally

[*]Corresponding authors:

Gunnar Mellgren, MD, PhD, Simon N. Dankel, PhD or Pierre Chymkowitch, PhD.

e-mails: gunnar.mellgren@uib.no, simon.dankel@uib.no or pierre.chymkowitch@ibv.uio.no

## Supplementary Figures

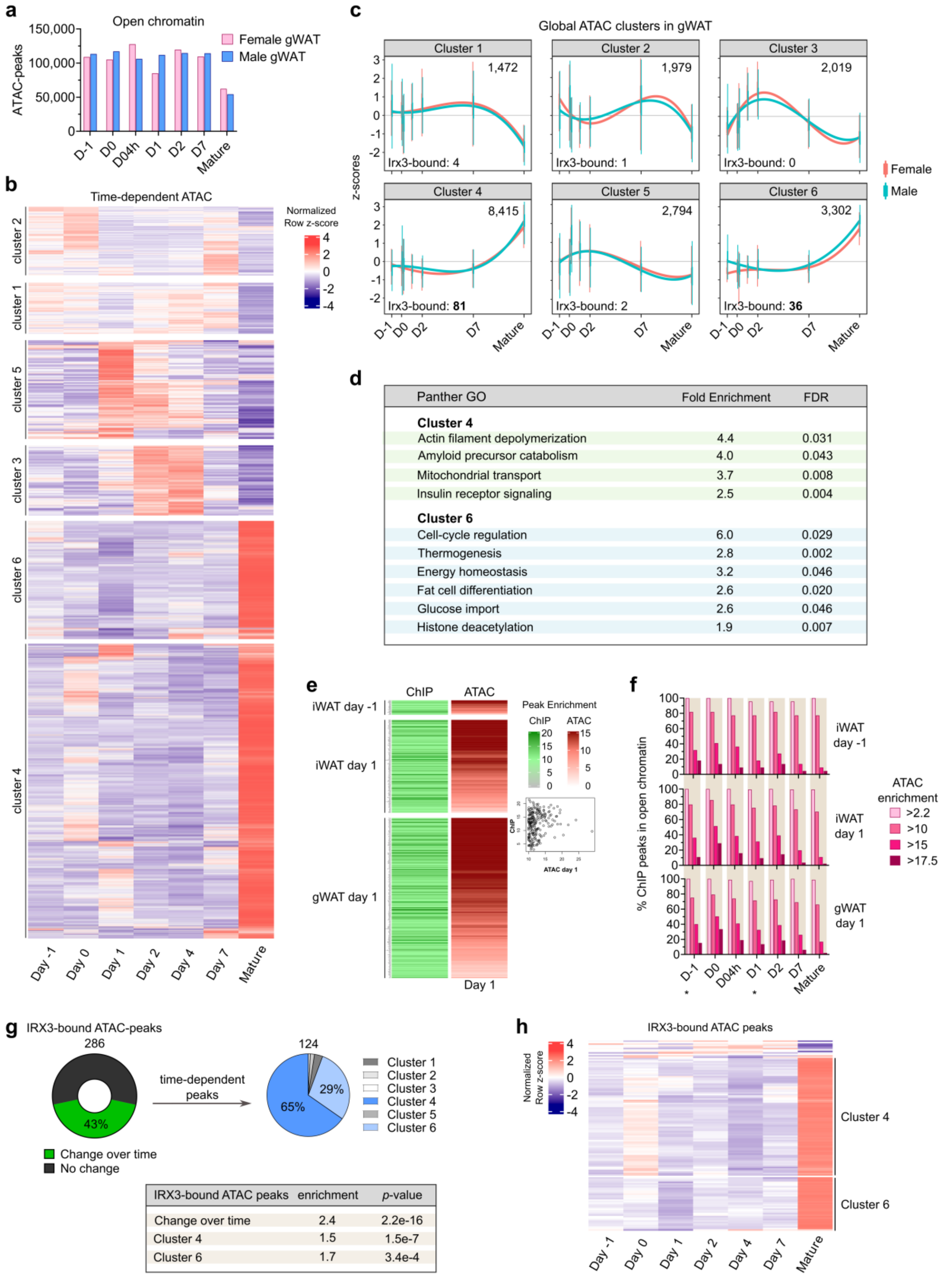


**Supplementary Fig. 1: IRX3 binds to open chromatin in gWAT.** Open chromatin profiling was performed by ATAC-sequencing at seven timepoints during differentiation of preadipocytes derived from gWAT of male and female mice (n = 2-3 samples, except for day -1 where n = 1). **a** Total number of ATAC peaks shown for each timepoint during gWAT differentiation. **b** Heatmap showing ATAC peaks with significant time-dependent change in magnitude. Regions with similar patterns of

openness over time are clustered according to maSigPro stepwise regression. Data presented as normalized row z-scores. **c** Average shape of each ATAC-seq cluster shown. The total number of ATAC peaks, as well as the number of IRX3-bound ATAC peaks is shown. **d** Selected Panther GOs of the two clusters comprising loci with increased chromatin opening during the last stages of differentiation. Fold enrichment and FDR shown. See also Supplementary Data 5 for all GOs from these two clusters. **e** Heatmap comparing enrichment of ChIP-seq peaks from all conditions (iWAT and gWAT) with ATAC-seq peaks in gWAT day 1. **f** Percentage of ChIP-seq peaks overlapping ATAC-seq peaks for each timepoint during differentiation. Asterisks denote the timepoints where ChIP-seq was performed and thereby are directly comparable with ATAC-status. Color of bars represent degree of ATAC-enrichment/chromatin openness. **g** Percentage of IRX3-bound ATAC peaks displaying changes over time (left). Distribution of IRX3-bound, time-dependent ATAC peaks according to the six global ATAC clusters (right). Enrichment of time-dependent IRX3-bound versus total ATAC-seq peaks shown (bottom). **h** Heatmap illustrating the high proportion of IRX3-bound, time-dependent ATAC peaks that belong to cluster 4 and 6; loci that display increased opening in mature adipocytes. Normalized row z-scores shown. See (b) for corresponding heatmap of total ATAC peaks.

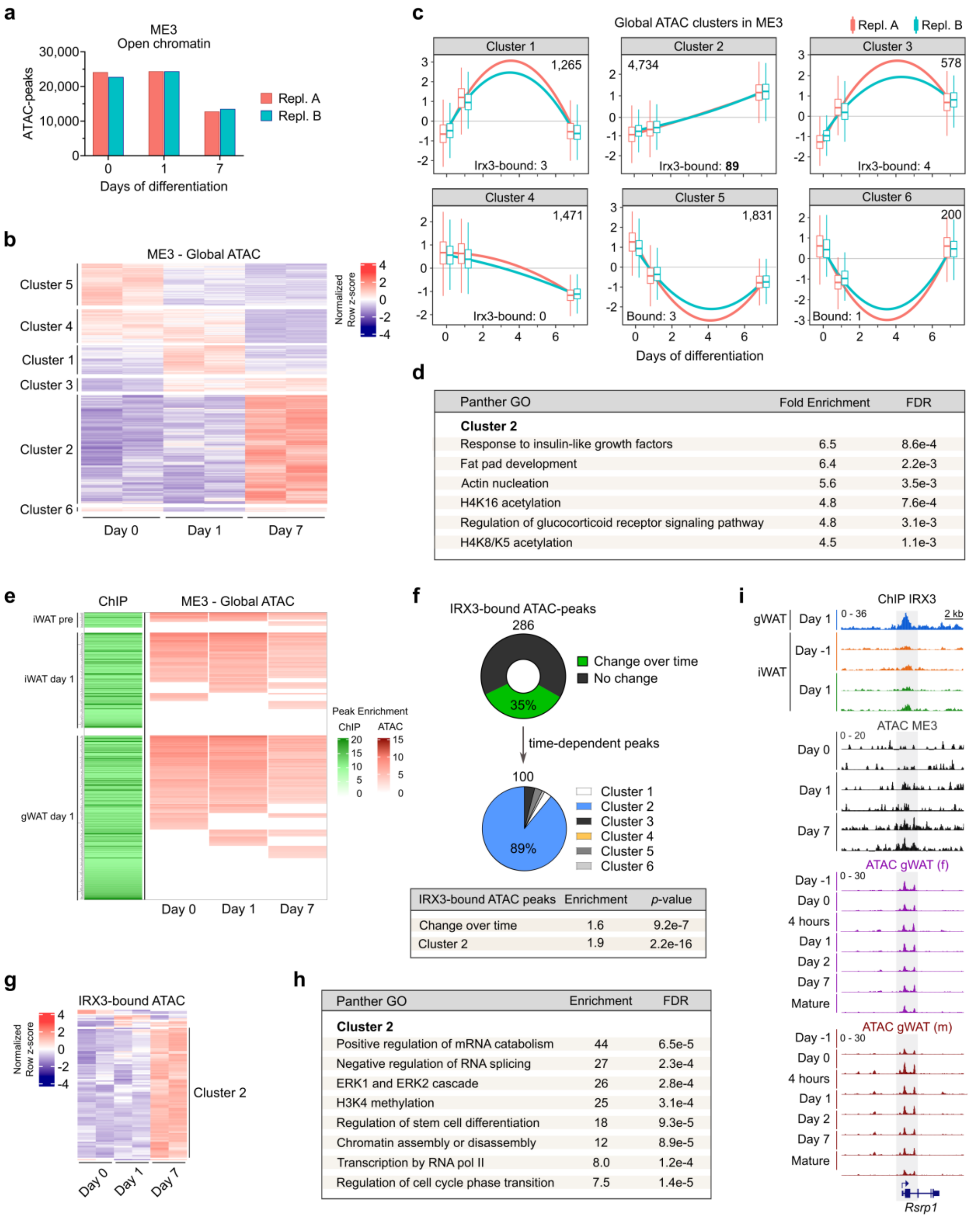


**Supplementary Fig. 2: IRX3 binds to open chromatin in beige ME3 cells.** Open chromatin profiling was performed by ATAC-sequencing at three timepoints during differentiation of beige ME3 preadipocytes (n = 2 biological replicates with 2 technical replicates each). **a** Total number of ATAC peaks shown for each timepoint during ME3 differentiation. **b** Heatmap of the ATAC peaks that displayed significant time-dependent change in magnitude. Normalized row z-scores shown. **c** Clustering of time-dependent ATAC peaks in ME3 according to maSigPro stepwise regression. The total number of ATAC peaks, as well as the number of IRX3-bound ATAC peaks shown. **d** Selected Panther GOs of cluster 2, comprising loci with increased chromatin opening during differentiation. Fold enrichment and FDR shown. See also Supplementary Data 5 for all GOs. **e** Heatmap comparing enrichment of ChIP-seq peaks from all conditions (iWAT and gWAT) with ATAC-seq peaks in ME3 days 0, 1 and 7. **f** Percentage of IRX3-bound ME3 ATAC peaks displaying changes over time (top). Distribution of IRX3-bound, time-dependent ATAC peaks in the six global ME3 ATAC clusters (middle). Enrichment of time-dependent IRX3-bound versus total ATAC-seq

peaks shown (bottom). **g** Heatmap illustrating the high proportion of IRX3-bound, time-dependent ME3 ATAC peaks that belong to cluster 2; loci that display increased opening in mature adipocytes. See (b) for corresponding heatmap of total ME3 ATAC peaks. **h** Panther GOs for genes bound by IRX3 and displaying increased chromatin opening during ME3 differentiation. **i** Genome browser tracks showing IRX3 occupancy at the *Rsrp1* promoter in iWAT and gWAT on days -1 and 1 and opening of chromatin during differentiation in the same locus in cells from gWAT (days -1, 0, 0+4h, 1, 2, 7 and in fully mature cells) and in ME3 cells (days 0, 1 and 7).

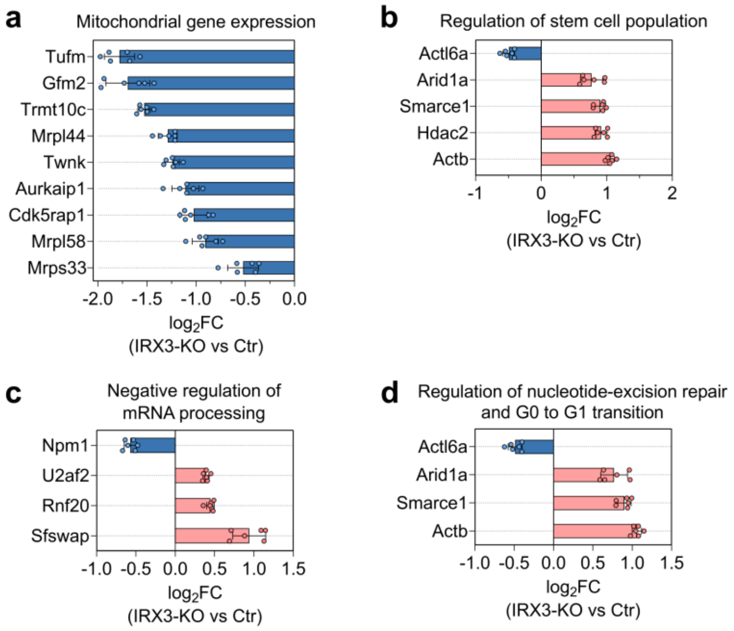


**Supplementary Fig. 3: Expression of direct IRX3 target genes in IRX3-KO cells from indicated GO categories on day 7 of differentiation in ME3 cells.** Log2 fold changes for each gene in IRX3-KO vs control cells for each of the following GO categories are shown (n = 6 replicate wells), data from [1]: **a** Mitochondrial gene expression, **b** Regulation of stem cell population, **c** Negative regulation of mRNA processing and **d** Regulation of nucleotide-excision repair and G0 to G1 transition. Downregulated genes, blue bars; upregulated genes, red bars. The bar graphs show means ± SD. Source data available in the Source Data file. Related to Fig. 2.

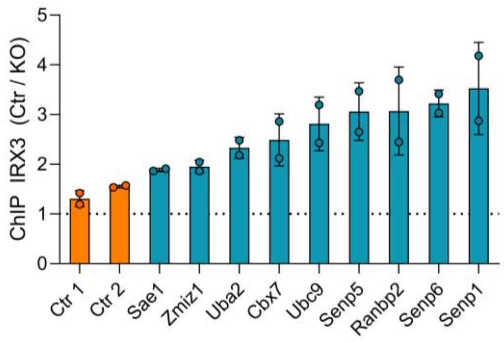


**Supplementary Fig. 4: IRX3 ChIP qPCR against SUMOylation pathway genes in ME3 cells.** ME3 control and IRX3-KO cells (n = 2 replicate wells) were differentiated until day 1 before formaldehyde fixation and chromatin isolation. Immunoprecipitation was performed using anti-IRX3 (ab25703), and qPCR was performed on input and immunoprecipitated DNA against the promoter region of genes in the SUMOylation pathway. Primer design was based on the IRX3 ChIP-seq peak locations in differentiating preadipocytes from iWAT and gWAT. The ratio of percent input in control over IRX3-KO is shown. Ctr1, Adipoq; Ctr2, mouse negative control primer set 1 (Active Motif) targeting a gene desert in chromosome 6. The bar graphs show means ± SD. Source data available in the Source Data file.

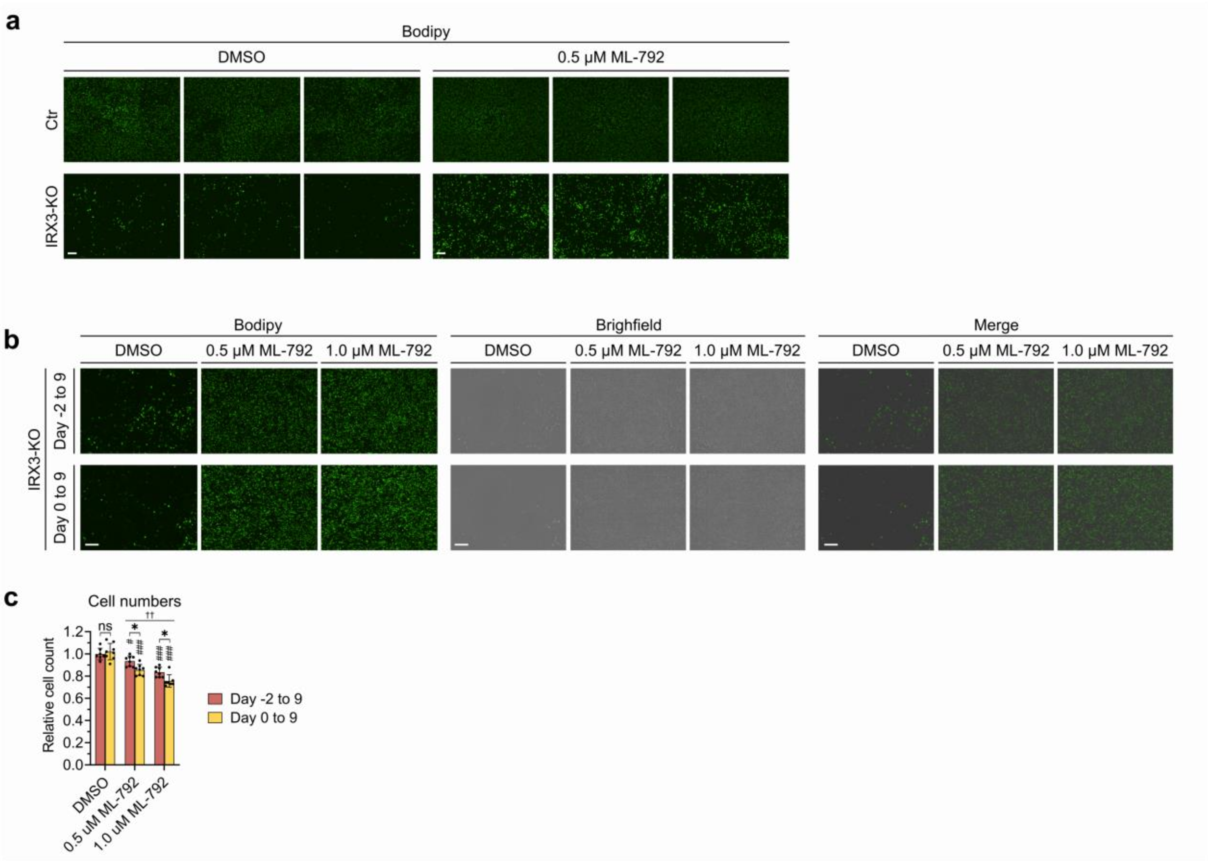


**Supplementary Fig. 5: Inhibition of SUMOylation partially restores adipogenesis in IRX3-KO ME3 cells. a** ME3 control and IRX3-KO cells were treated with either vehicle or the SUMOylation inhibitor ML-792 from days -2 to 9 of adipogenic differentiation. On day 9, the cells were treated with the Bodipy fluorescent lipid stain for 1 hour and imaged with fluorescence microscopy. Nine images/well were stitched to represent each well of a 24-well plate. Bodipy signal in n = 3 replicate wells from one of four independent experiments is shown. Scale bar = 400 µm. **b** Dose-response effect of ML-792 on lipid accumulation in IRX3-KO cells when 0.5 µM or 1.0 µM ML-792 was added on either day -2 or 0 and kept throughout differentiation until day 9. Four images from each well of a 96-well plate were stitched together. One representative well from one of three independent experiments is shown with Bodipy fluorescent signal (left), brightfield (middle) and merge (right). Scale bar = 400 µm. **c** Dose-response effect of 0.5 µM and 1.0 µM ML-792 on relative cell count in IRX3-KO cells. n = 8 replicate wells from one of three independent experiments. $^{*}p_{adj} = 0.02$ (effect of days); $^{\#}p_{adj} = 0.03$, $^{\#\#\#}p_{adj} < 0.001$ (ML-792 vs DMSO); $^{\dagger\dagger}p_{adj} = 0.001$ (0,5 µM vs 1.0 µM ML-792); two-way ANOVA with Holm–Sidak correction. The bar graphs show means ± SD. Source data available in the Source Data file. Related to Fig. 4.

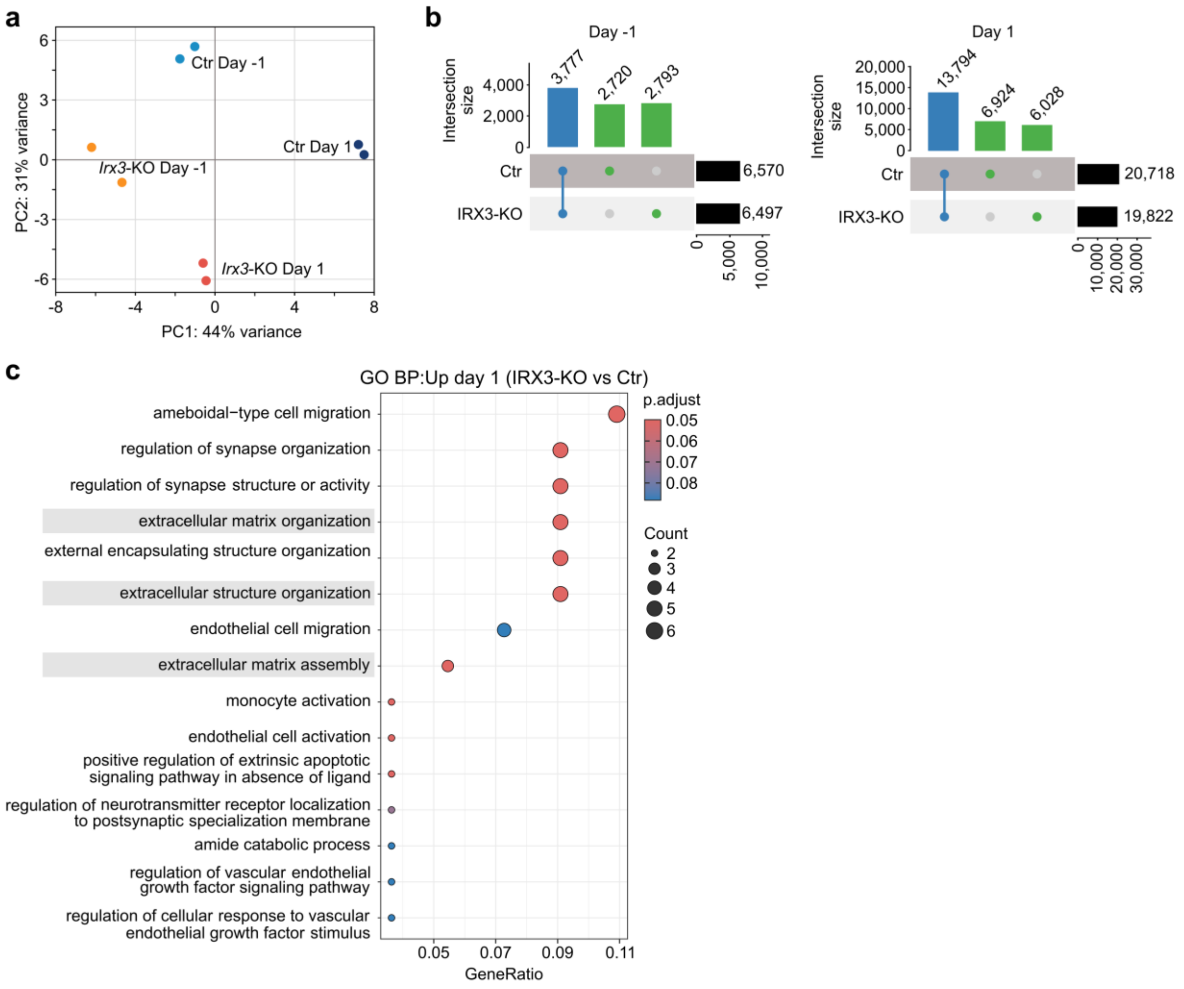


**Supplementary Fig. 6: SUMO2/3 ChIP-seq.** ME3 control and IRX3-KO cells were subjected to adipogenic differentiation and fixated on days -1 and 1 (n = 2 replicates), followed by chromatin extraction, immunoprecipitation by a SUMO2/3 antibody (ab3742) and sequencing. **a** PCA plot showing the relative contribution of replicates, genotype and timepoint to the observed variation. **b**Upset plots showing the number of common and unique SUMO2/3 peaks in control and IRX3-KO cells. Black, blue and green bars represent the total number of peaks in each sample, common peaks and unique peaks, respectively. **c** Gene enrichment analysis showing the top 15 most significant GO terms for hyperSUMOylated loci in IRX3-KO vs control cells on day 1 of adipogenic differentiation. For complete lists of GO terms, see Supplementary Data 8. Source data available in the Source Data file. Related to Fig. 6.

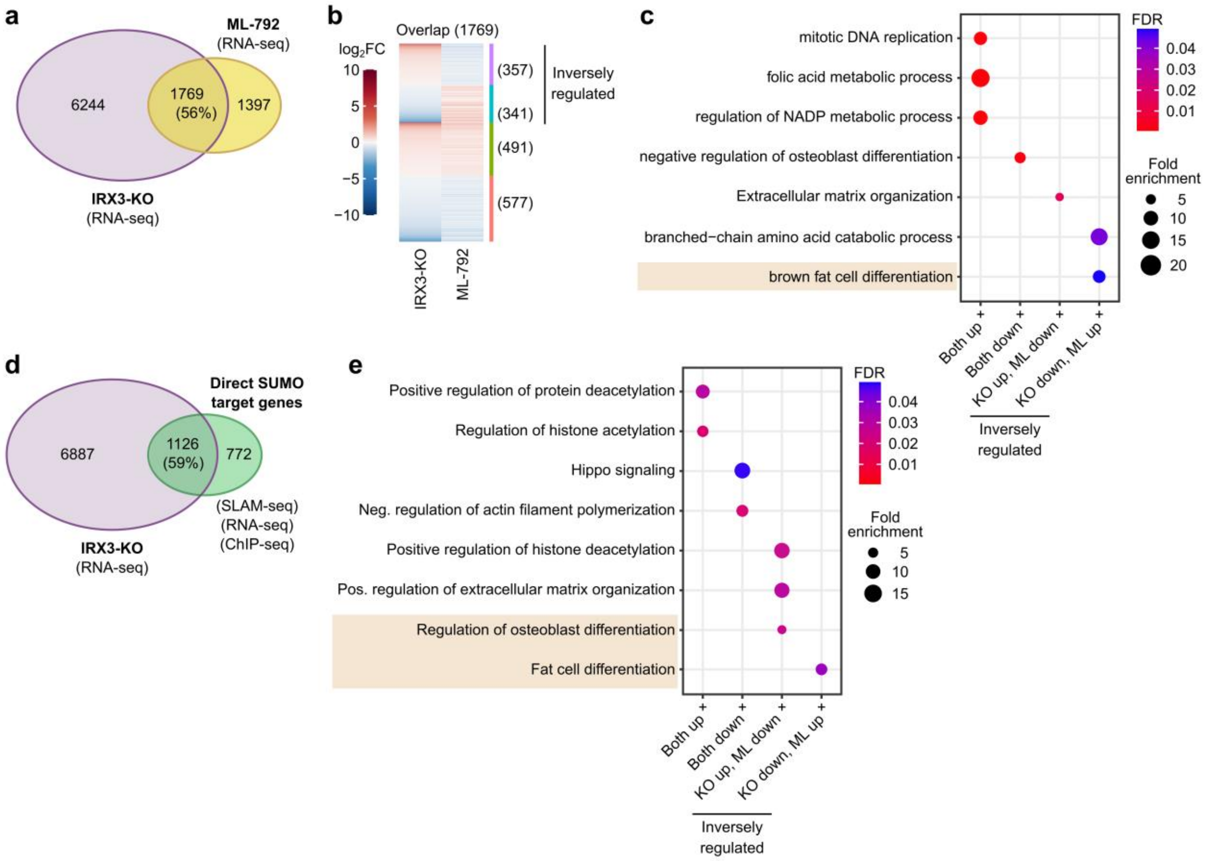


**Supplementary Fig. 7: IRX3 and SUMO share common target genes involved in adipocyte versus osteoblast development on day 1 of adipogenesis in 3T3-L1 and ME3 cells.** Global mRNA-sequencing was performed to map DEGs between ME3 control and IRX3-KO cells on day 1 of differentiation ($p_{adj}$ <0.01, FC >1.2; n = 6; data from [1]) and either DEGs between 3T3-L1 cells treated with vehicle or 0.5 µM ML-792 on day 1 of differentiation (FC ≥1.2 and $p_{adj}$. ≤0.001, (n = 3, data from [2]) alone or in combination with a list of *newly transcribed* genes as measured by SLAM-seq ($p_{adj}$ ≤0.05, FC ≥1.2, n = 3, data from [2]) and a SUMO-ChIP-seq dataset genes (n = 2, data from [2]) to make a list of direct SUMO target genes which in turn was compared with the list of IRX3-responsive genes. **a** Venn diagram showing overlap between DEGs in response to IRX3-KO in ME3 cells and ML-792 in 3T3-L1 cells, both on day 1 of differentiation and measured by mRNA-seq. **b** Heatmap showing direction of change among overlapping genes in (a). **c** Selection of top enriched/most significant GO categories among the overlapping genes in (a-b). See Supplementary Data 11 for complete lists. **d** Venn diagram showing overlap of DEGs in ME3 control versus IRX3-KO cells on day 1 of differentiation with direct SUMO target genes on day 1 of differentiation in 3T3-L1 cells. See also Supplementary Data 12. **e** Selection of top enriched/most significant GO categories among the overlapping genes in (d). See Supplementary Data 12 for complete lists. Source data available in the Source Data file.

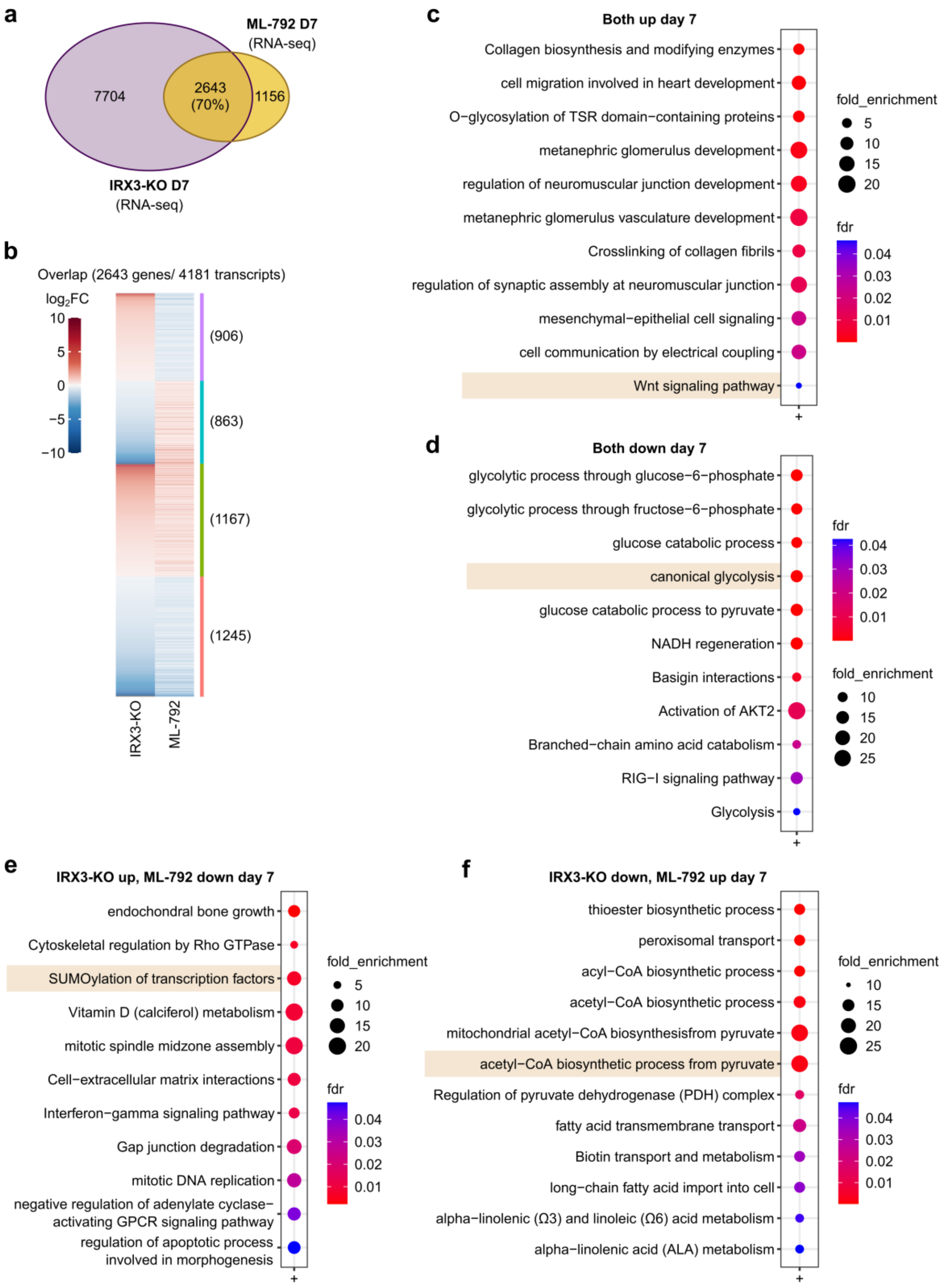


**Supplementary Fig. 8: Overlap between IRX3 and SUMO target genes on day 7 of adipogenesis in ME3 and 3T3-L1 cells**
Global mRNA-sequencing was performed to detect differentially expressed genes (DEGs) between ME3 control and IRX3-KO cells on day 7 of differentiation (FC ≥1.2 and $p_{adj}$. ≤0.001, n = 6, data from [1]) and DEGs between 3T3-L1 cells treated with vehicle or 0.5 µM ML-792 on day 7 of differentiation (FC ≥1.2 and $p_{adj}$. ≤0.001, n = 3, data from [2]). **a** Venn diagram showing overlap between DEGs following IRX3-KO or ML-792 on day 7 of differentiation. **b** Heatmap showing direction of change among overlapping genes in (a). **c**-**f** Selection of top enriched/most significant GO categories among the overlapping genes in (a-b). GO terms for upregulated (**c**), downregulated (**d**), and inversely regulated (**e**-**f**) genes between the two datasets shown. Source data available in the Source Data file.

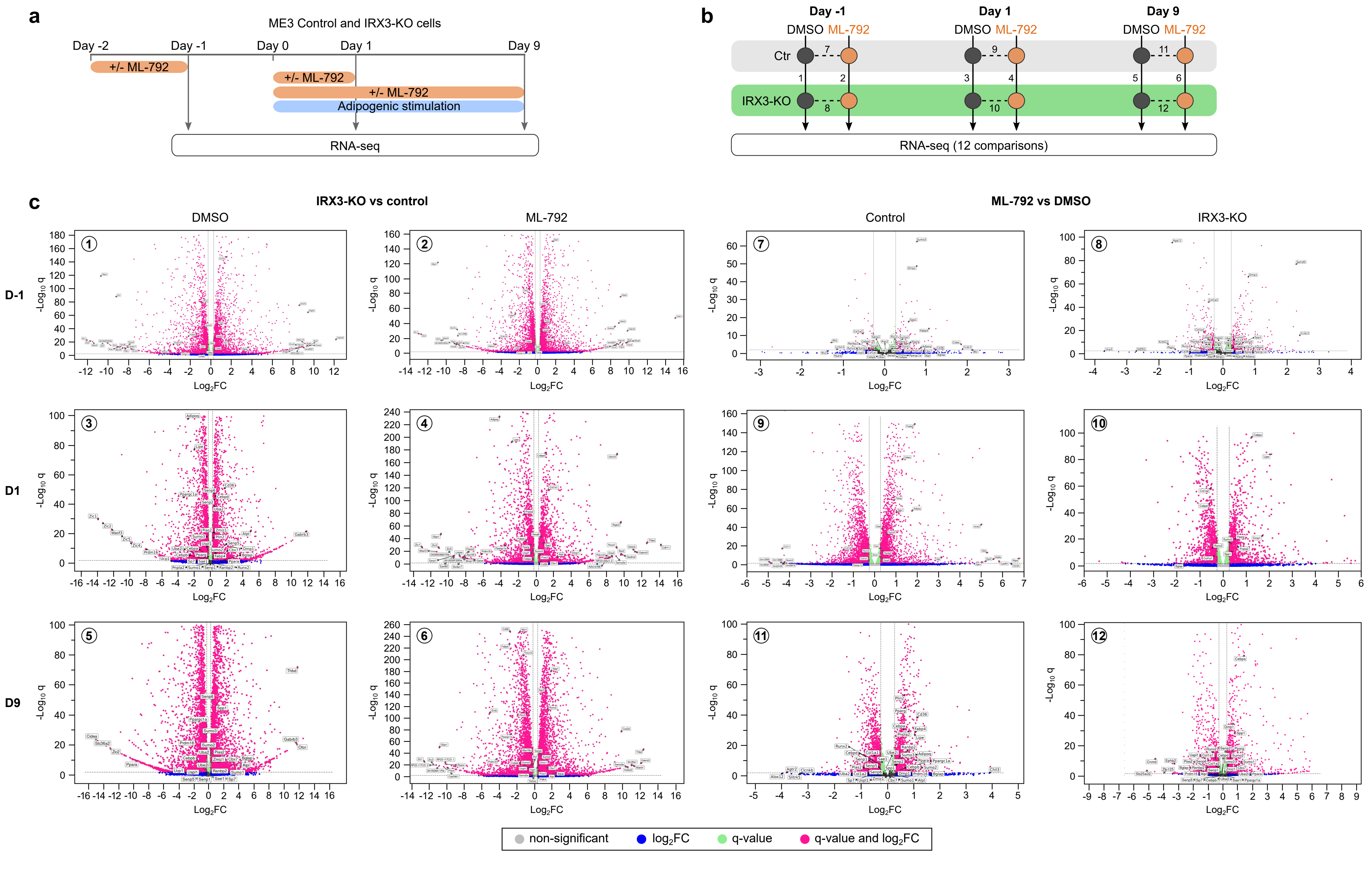
a
ME3 Control and IRX3-KO cells
Day -2
Day -1
Day 0
Day 1
Day 9
+/- ML-792
+/- ML-792
+/- ML-792
Adipogenic stimulation
RNA-seq
b
Day -1
Day 1
Day 9
DMSO ML-792
Ctr
IRX3-KO
RNA-seq (12 comparisons)
c
IRX3-KO vs control
ML-792 vs DMSO
DMSO
ML-792
Control
IRX3-KO
D-1
D1
D9
-Log10 q
Log2FC
non-significant
log2FC
q-value
q-value and log2FC

**Supplementary Fig. 9: Differential gene expression following IRX3-KO and/or ML-792 treatment in ME3 cells.** ME3 control and IRX3-KO cells were treated with DMSO or 0.5 µM ML-792 before and at different stages of adipogenic differentiation (n =3 replicate wells for each condition). RNA was collected on days -1, 1 and 9 and differences in gene expression analyzed by RNA-seq. **a** Schematic illustrating the time and duration of the DMSO or ML-792 treatment, as well as the days of RNA collection. Cells collected on days -1 and 1 of adipogenic differentiation had received DMSO or ML-792 treatment for 24h, while cells collected at day 9 had received DMSO or ML-792 treatment for 9 days. **b** Detailed schematic of the experimental design. Days indicate the day of cell harvest; each dot represents an experimental condition with n = 3 replicate wells; black and orange dots represent DMSO and ML-79 treatment, respectively. Solid lines indicate IRX3-KO vs control comparisons; dashed lines represent ML-792 vs DMSO. Numbers 1-12 denote each pairwise comparison. **c** Volcano plots for each of the 12 comparisons shown in (B). Pink dots represent significantly ($q < 0.01$) differentially expressed genes ($log_2FC > 0.265$ or $< -0.265$).

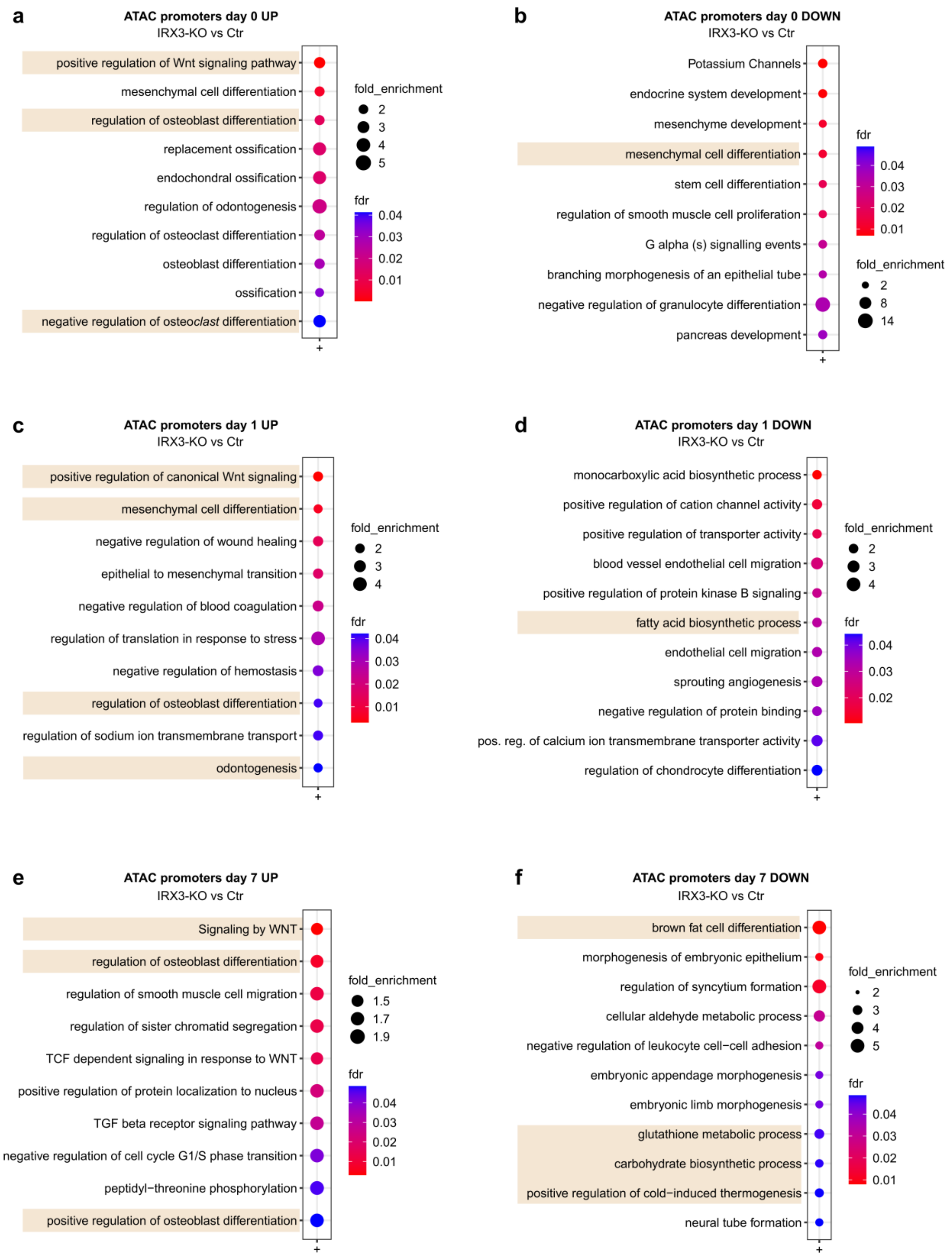


**Supplementary Fig. 10: GOs for differential ATAC-seq peaks in promoters of IRX3-KO vs control cells.** ME3 control and IRX3-KO cells were treated with adipogenic stimuli for 7 days, and open chromatin profiling was performed on days 0, 1 and 7 by ATAC-seq (n = 3 replicate wells per condition). The most strongly enriched and/or significant GO terms for genes with changes in open chromatin in their promoter on day 0 (**a**-**b**), day 1 (**c**-**d**) and day 7 (**e**-**f**) is shown. More open (**a, c, e**) and less open **(b, d, f)** indicated. See Supplementary Data 18 for complete lists. Source data available in the Source Data file**.**

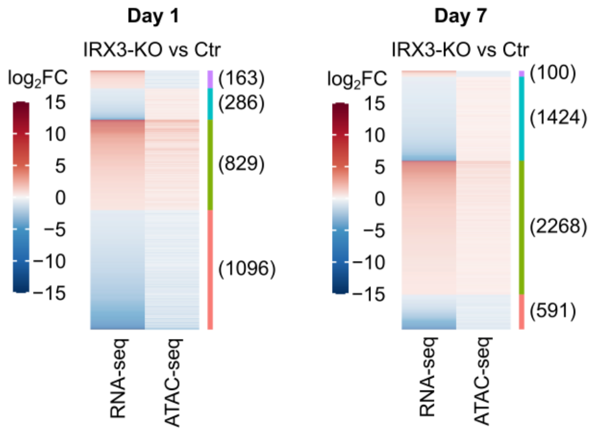


**Supplementary Fig. 11: Changes in open chromatin and gene expression following IRX3-KO.**
Global changes in open chromatin in promoters of IRX3-KO vs control ME3 cells was measured by ATAC-seq (n = 3 replicate wells) and compared with changes in gene expression by RNA-seq (n = 6 replicate wells, data from [1]). $Log_2$ fold changes on day 1 (left) and day 7 (right) is shown.

**Supplementary references**

[1] Bjune, J.-I., Dyer, L., Røsland, G.V., Tronstad, K.J., Njølstad, P.R., Sagen, J.V., et al., 2020. The homeobox factor Irx3 maintains adipogenic identity. Metabolism: Clinical and Experimental 103.

[2] Zhao, X., Hendriks, I.A., Le Gras, S., Ye, T., Ramos-Alonso, L., Aur´, A., et al., 2022. Waves of sumoylation support transcription dynamics during adipocyte differentiation. Nucleic Acids Research 50(3): 1351–69.

# Sequencing project S24137

## 1 General information about the project

Application: **ChIP-seq**
Library preparation method: **Library prep ChIP (Diagenode)**
Sequencing type: **Paired-End**[1]
Read length: **2 X 50**
Sequencer: **Illumina NextSeq 2000**
Organism: **Mus musculus**
Library information:

- Library name : **P3212**
  - Reference : Diagenode MicroPlex Library Preparation kit v3 Instruction Manual
  - Version : v.3 Jun 2021 (a description of this protocol is available in "Publication" section)

Table 1: **Information about the samples.**

| Sample ID | Sample name | Library number | Index used to demultiplex | Flowcell | Lane |
|---|---|---|---|---|---|
| PRCH577 | In1 | P3212 | CAAGCTAG / ACATAGCG | FC24109 | L1 |
| PRCH578 | In2 | P3212 | TGGATCGA / GTGCGATA | FC24109 | L1 |
| PRCH579 | IP1 | P3212 | GGCTTAAG / TCGTGACC | FC24109 | L1 |
| PRCH580 | IP2 | P3212 | AATCCGGA / CTACAGTT | FC24109 | L1 |
| PRCH581 | IP3 | P3212 | GCAGAATT / ACCGGCCA | FC24109 | L1 |
| PRCH582 | IP4 | P3212 | ATGAGGCC / GTTAATTG | FC24109 | L1 |
| PRCH583 | IP5 | P3212 | ACTAAGAT / AACCGCGG | FC24109 | L1 |
| PRCH584 | IP6 | P3212 | GTCGGAGC / GGTTATAA | FC24109 | L1 |
| PRCH585 | IP7 | P3212 | TACCGAGG / CCTGAACT | FC24109 | L1 |
| PRCH586 | IP8 | P3212 | CGTTAGAA / TTCAGGTC | FC24109 | L1 |

Table 1 provides some information about the samples.

## 2 Number of sequenced reads

1 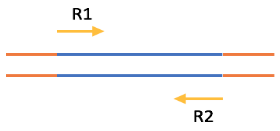

R1 (read 1) and R2 (read 2), in yellow, correspond to forward and reverse read, respectively. Adaptors are represented in orange and fragment in blue.

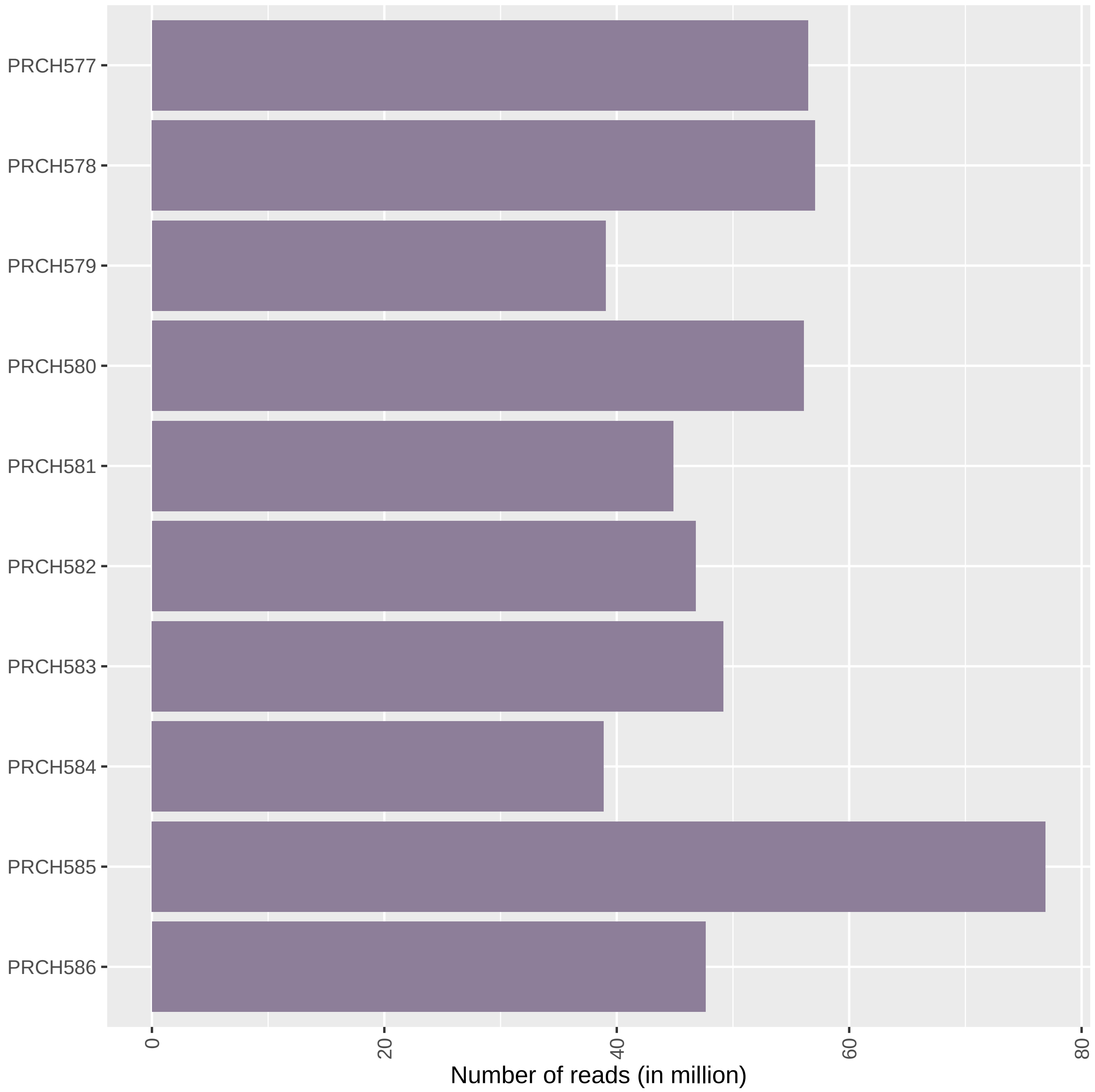


Figure 1: **Number of sequenced reads.** This barplot represents the total number of sequenced reads (in million).

Table 2: **Number of sequenced reads.**

| Sample ID | Sample name | Number of reads |
|---|---|---|
| PRCH577 | In1 | 56,465,227 |
| PRCH578 | In2 | 57,065,624 |
| PRCH579 | IP1 | 39,043,854 |
| PRCH580 | IP2 | 56,087,569 |
| PRCH581 | IP3 | 44,857,323 |
| PRCH582 | IP4 | 46,779,892 |
| PRCH583 | IP5 | 49,170,098 |
| PRCH584 | IP6 | 38,871,532 |
| PRCH585 | IP7 | 76,890,522 |
| PRCH586 | IP8 | 47,632,627 |
| Total number of reads | | 512,864,268 |
| Number of expected reads | | 400,000,000 |

As paired-end sequencing was performed, the term "read" refers to a sequenced fragment. Therefore all numbers indicated in this document have to be multiplied by two to obtain the number of sequences.

Figure 1 on the previous page and table 2 provide the number of reads in each sample. For samples sequenced on several lanes, FASTQ files presented in this report correspond to the concatenation of reads from all lanes.

## 3 Sequences quality controls

All quality controls presented in this section have been computed using FASTQC [1].

Figure 2 on the next page provides the percentage of bases with a quality score above 30 (corresponding to an error probability of 1/1000).

Figures 3 on page 5 to 4 on page 6 represent the average of the quality score at each position in each sample, for read 1 and read 2.

Figures 5 on page 7 to 6 on page 8 provide the percentage of each base (A, C, G and T) at each position along the read, for read 1 and read 2.

Figures 7 on page 9 to 8 on page 10 represent the percentage of duplicated reads based on the first 50bp of the first 200,000 reads in function of their duplication level, for read 1 and read 2.

Figures 9 on page 11 to 10 on page 12 provide the percentage of different[2] reads in each sample, for read 1 and read 2.

## 4 Library screen

Figures 11 on page 13 to 12 on page 14 provide the proportion of reads aligned to each DNA sequence. FastQScreen [2] and [3] was used to map a subset of reads (10,000,000) from each sample to selected DNA sequences in order to assess the presence of contamination within the samples.

## 5 Available files

[2]For a given sample, the set of different reads contains all distinct reads, whatever their occurrence number. For instance, for the following set of reads $\{A, B, C, C, D, E, F, F, F, G\}$, the set of different reads is $\{A, B, C, D, E, F, G\}$.

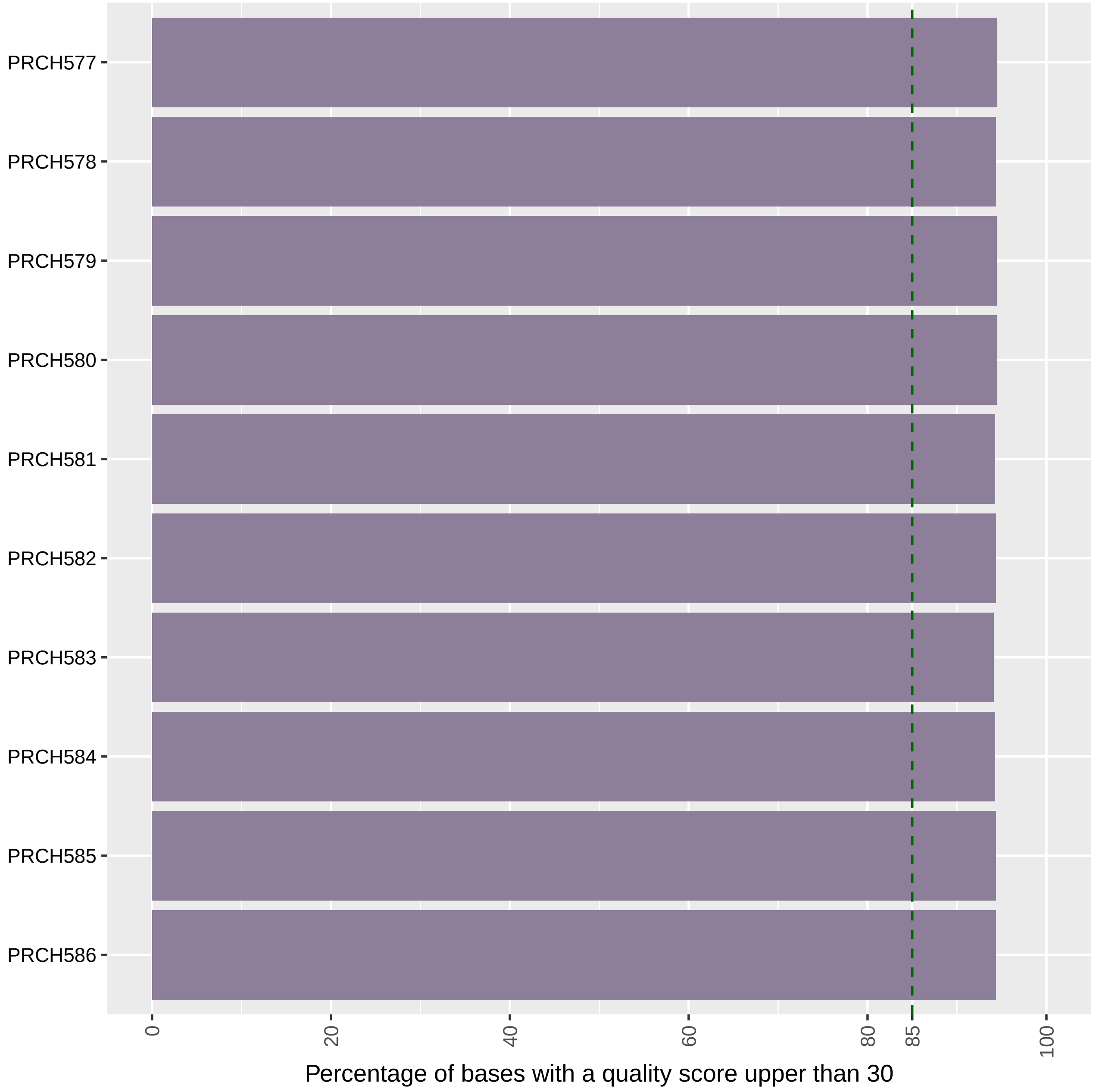


Figure 2: **Base quality in all samples.** This barplot represents the percentage of bases with a quality score per base upper than 30. Our quality threshold is represented with a dashed dark green line. If a sample does not reach the threshold, its name is represented in red color.

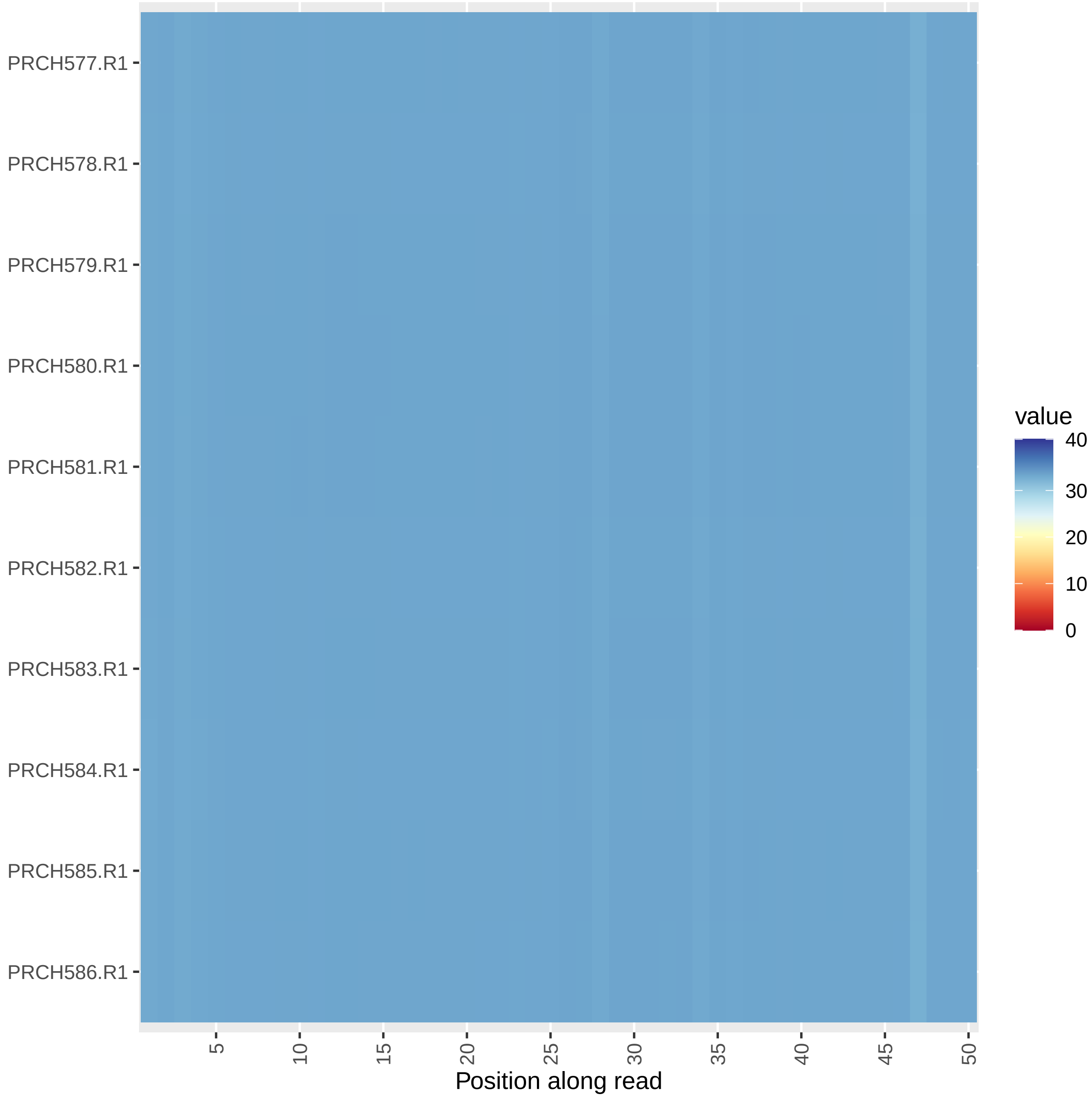


Figure 3: **Base quality along read 1 in all samples.** The mean quality score at each read position in each sample is represented on this heatmap. A quality score of 20 and 30 represents an error rate of 1/100 and 1/1000, respectively.

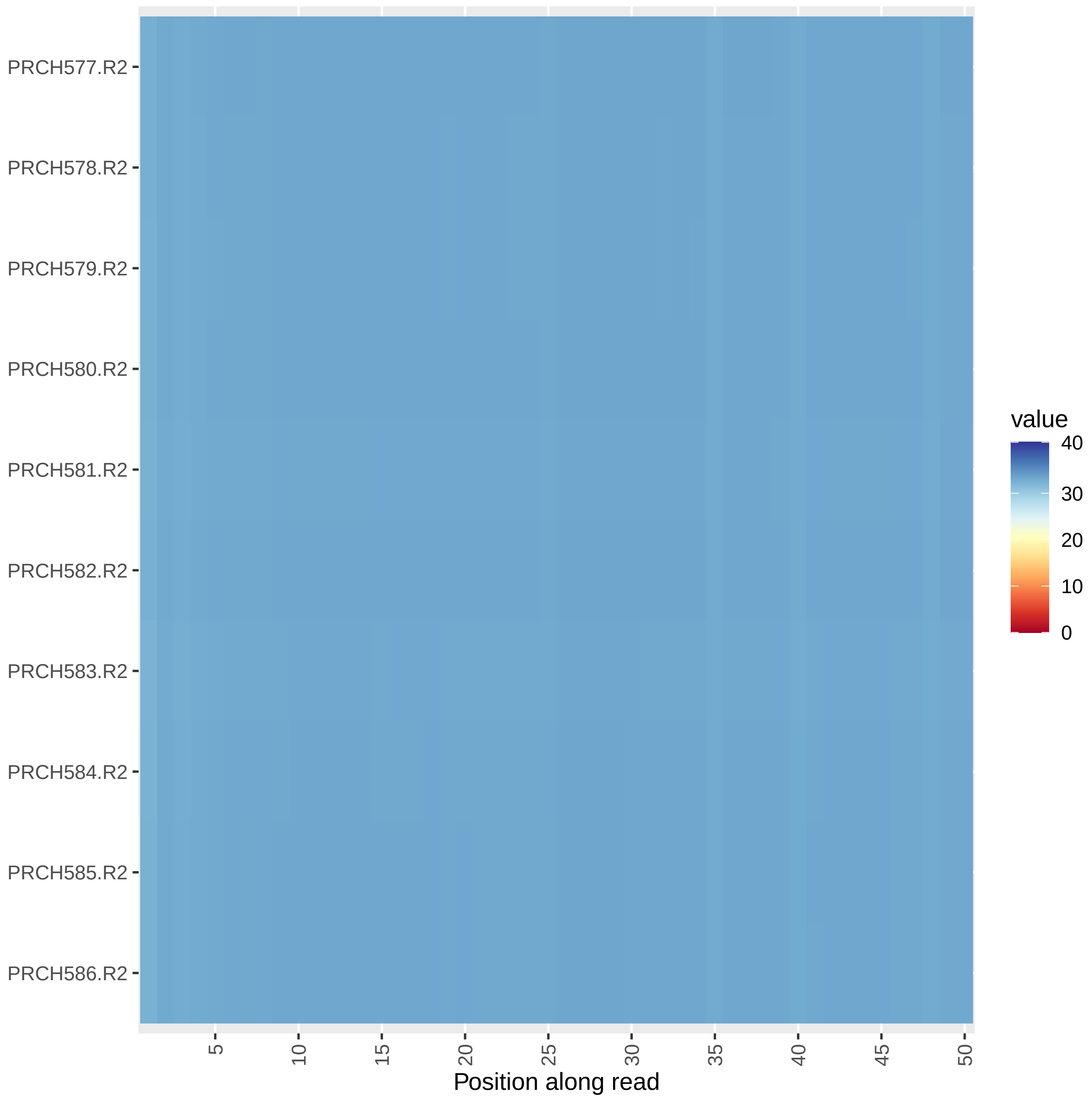


Figure 4: **Base quality along read 2 in all samples.** The mean quality score at each read position in each sample is represented on this heatmap. A quality score of 20 and 30 represents an error rate of 1/100 and 1/1000, respectively.

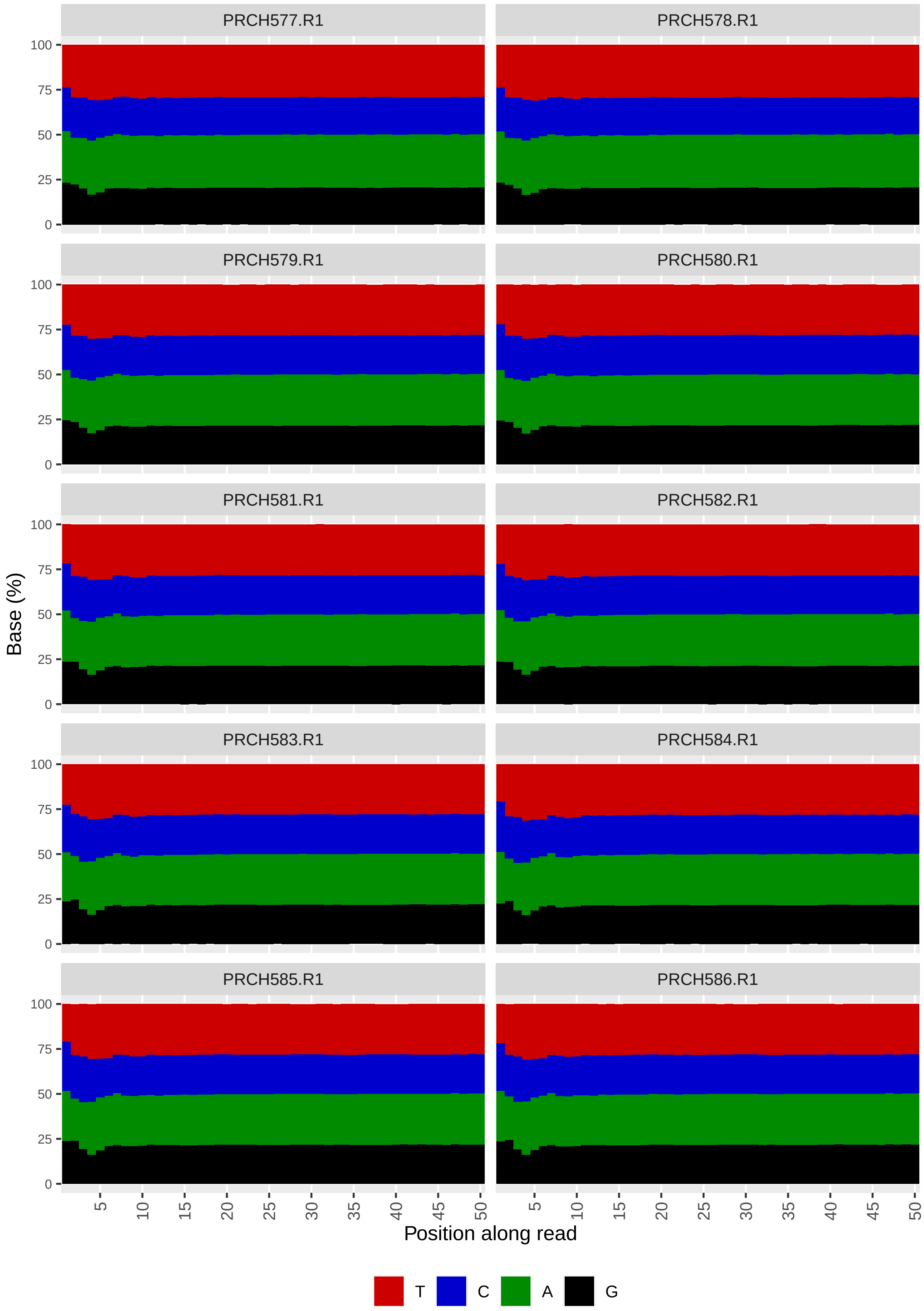


Figure 5: **Read 1 base sequence content.** The percentage of each base at each read position is represented on this figure.

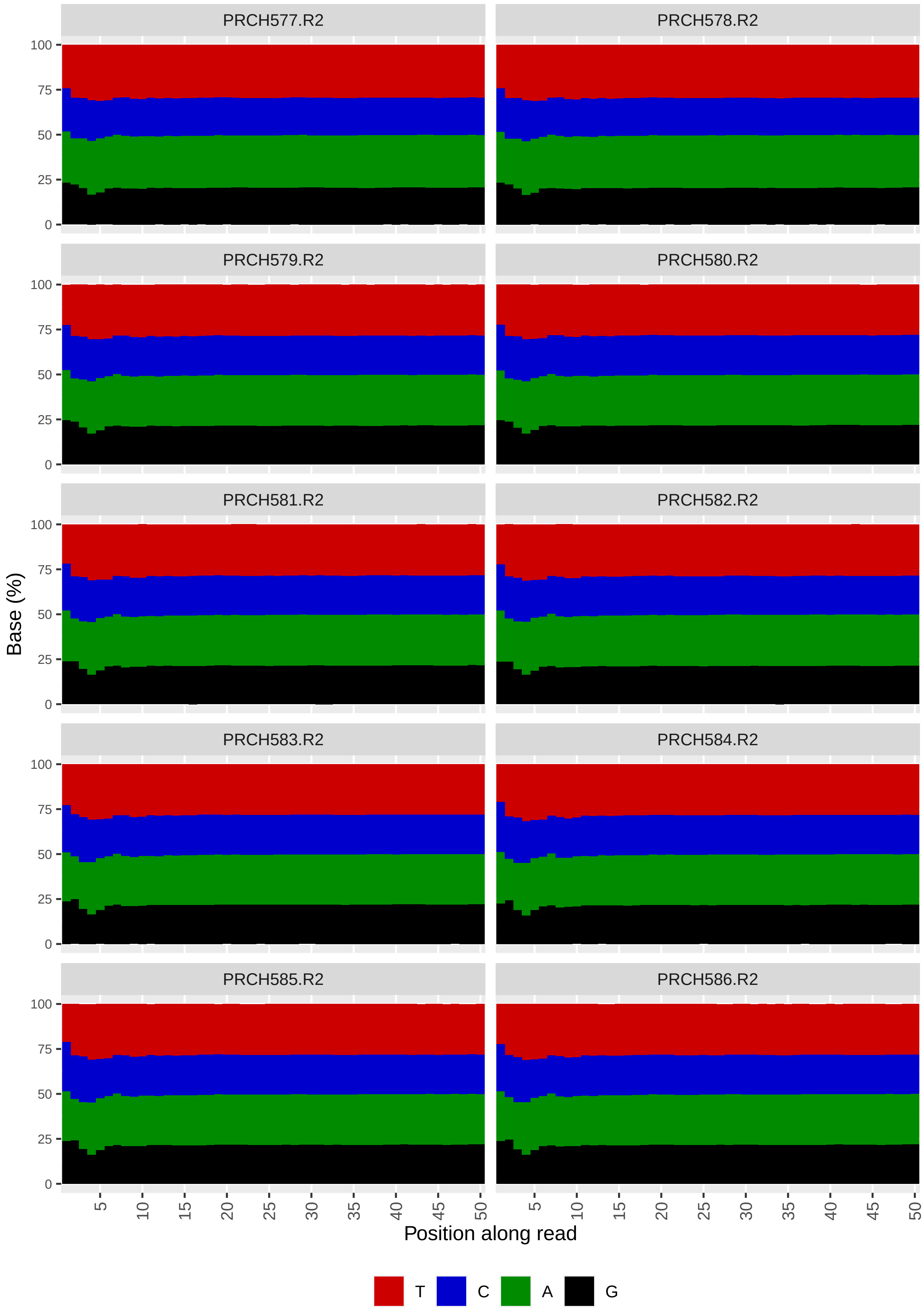


Figure 6: **Read 2 base sequence content.** The percentage of each base at each read position is represented on this figure.

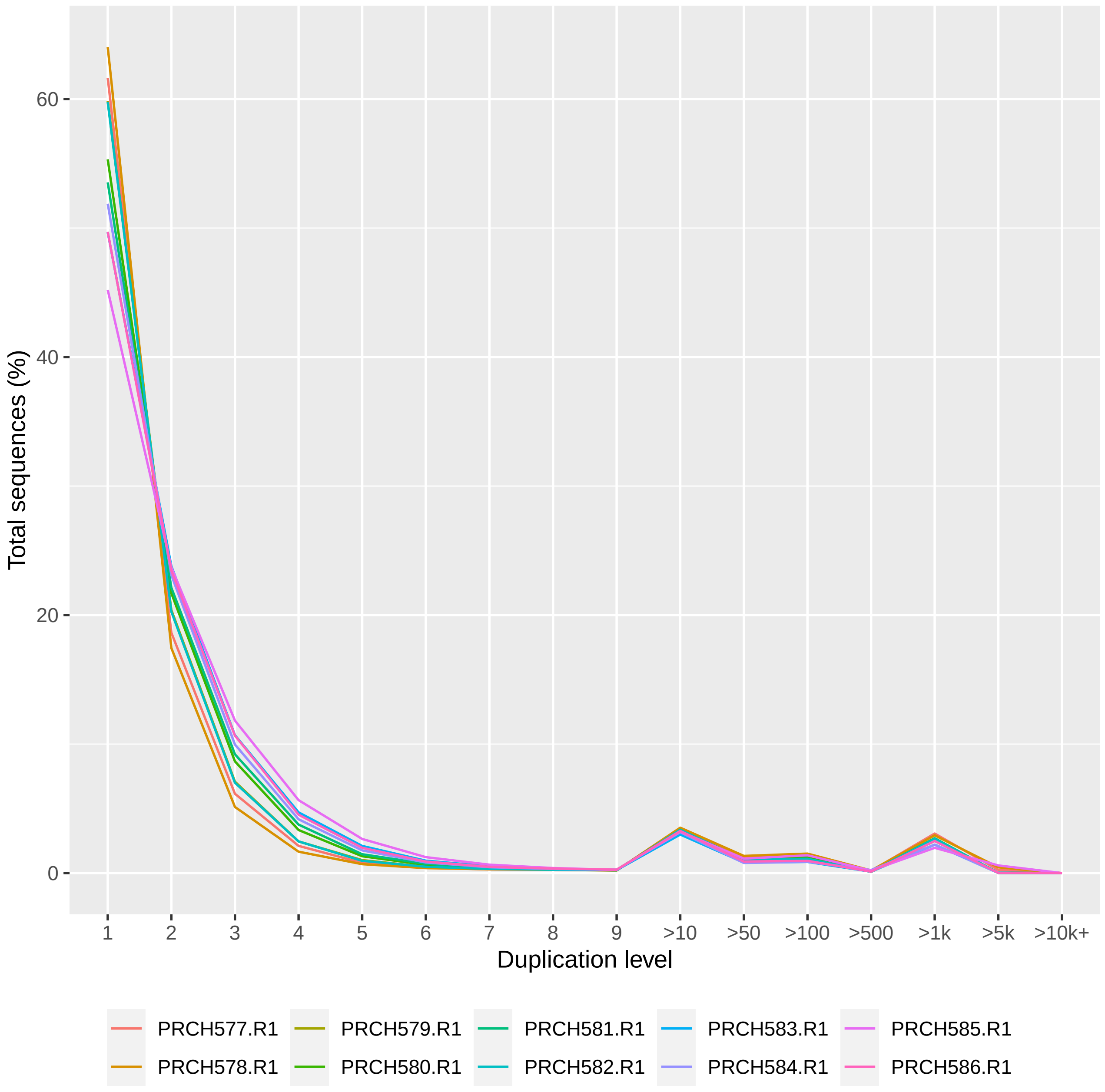


Figure 7: **Percentage of duplicated read 1.** The x-axis represents the duplication level and the y-axis is the percentage of total sequences. A duplication level of 1 means that the read is only seen one time in the set of reads, i.e. it represents the proportion of unique reads; a duplication level of 2 means that a given read is seen two times in the set of reads, and so on. For a given duplication level, the line represents the proportion of all sequences relative to the total number of reads.

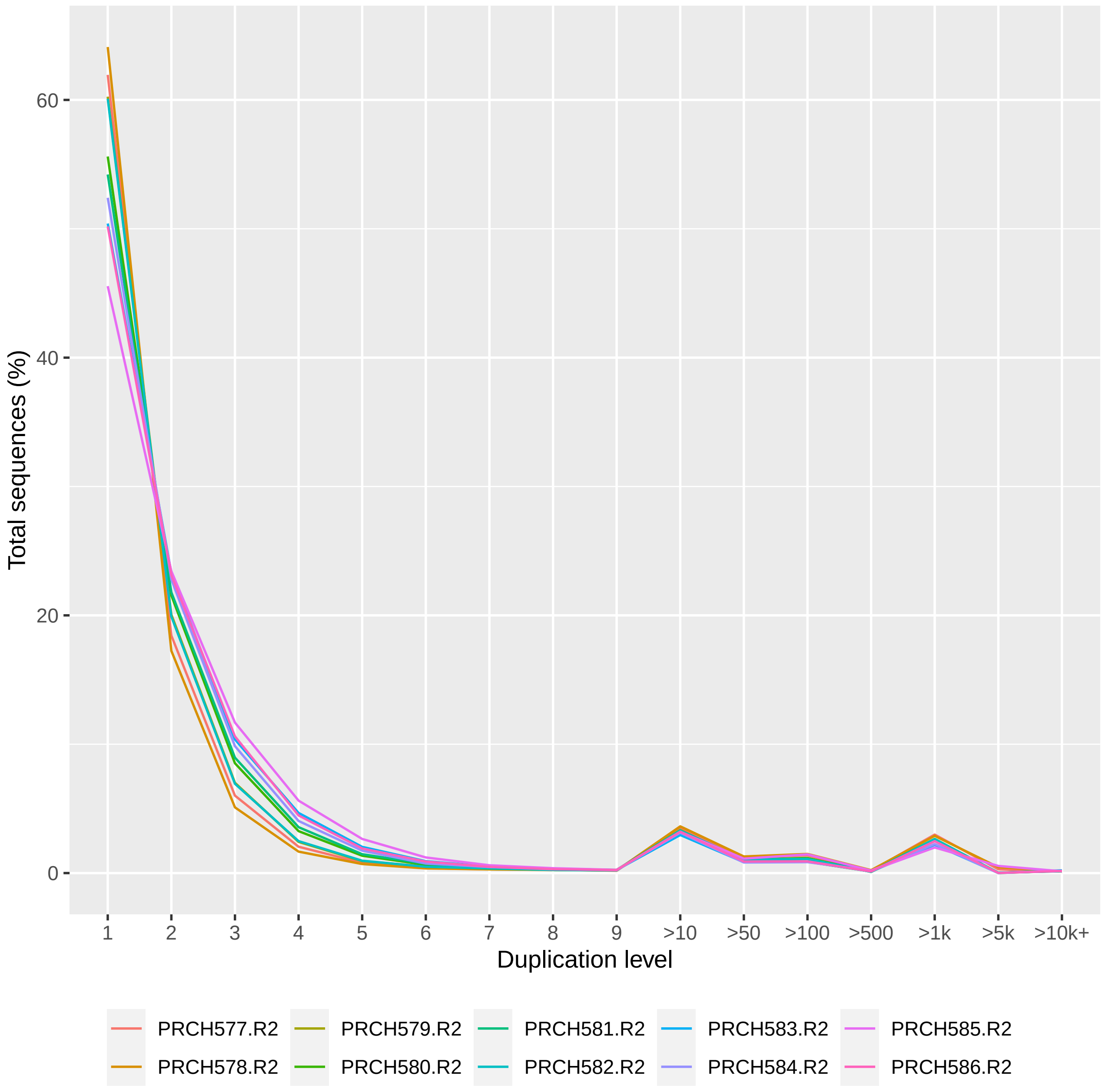


Figure 8: **Percentage of duplicated read 2.** The x-axis represents the duplication level and the y-axis is the percentage of total sequences. A duplication level of 1 means that the read is only seen one time in the set of reads, i.e. it represents the proportion of unique reads; a duplication level of 2 means that a given read is seen two times in the set of reads, and so on. For a given duplication level, the line represents the proportion of all sequences relative to the total number of reads.

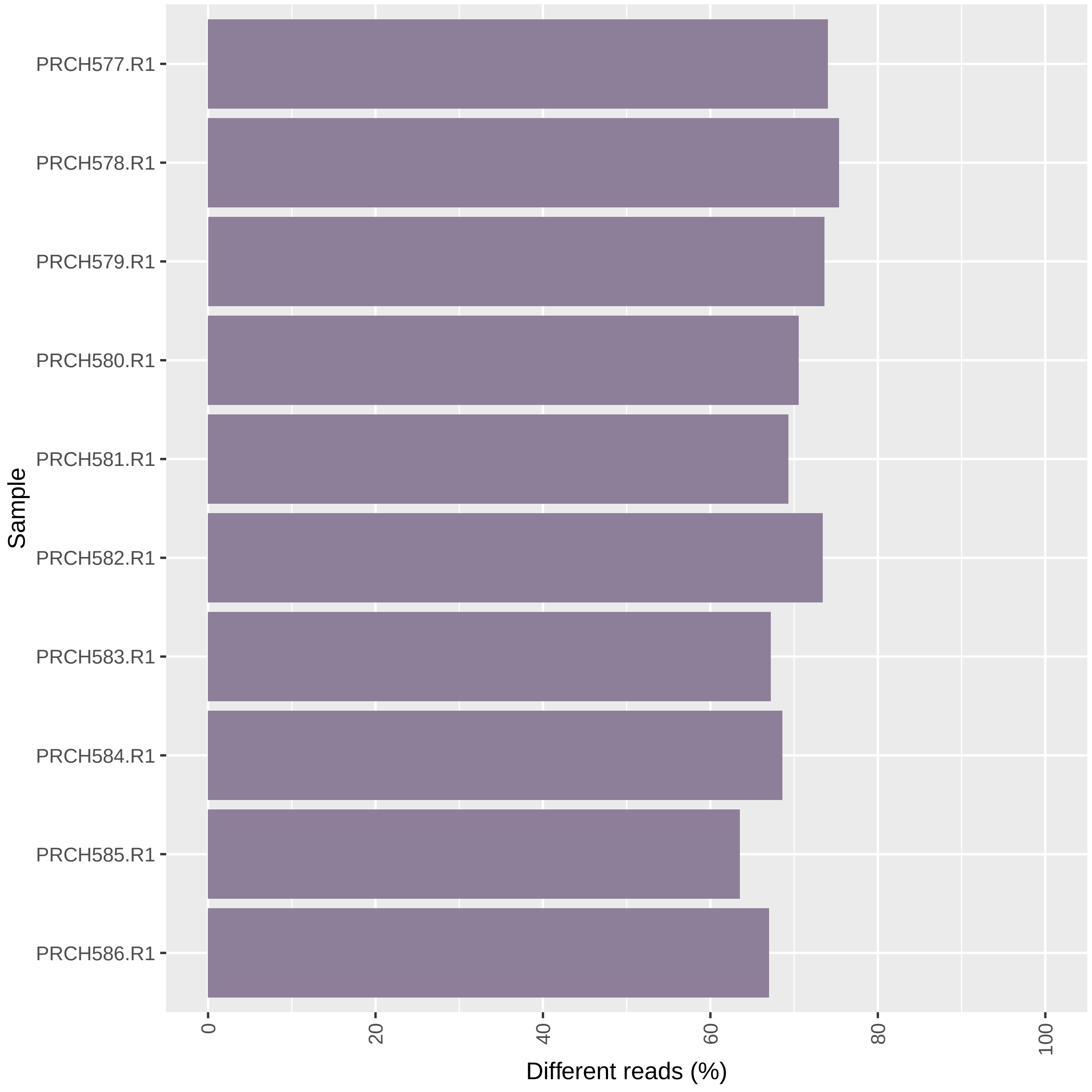


Figure 9: **Different read 1.** This figure represents the percentage of different read 1 in each sample.

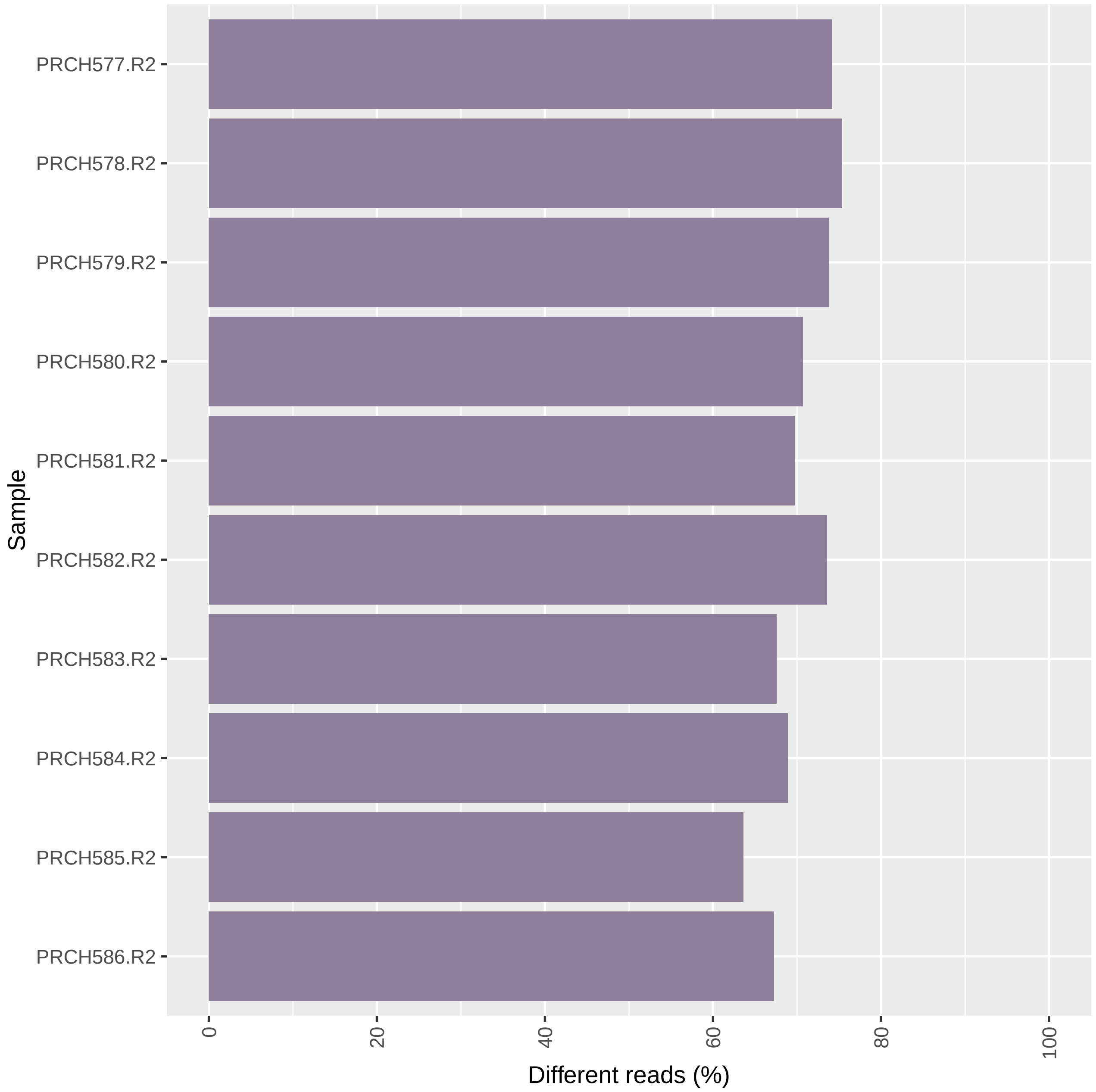


Figure 10: **Different read 2.** This figure represents the percentage of different read 2 in each sample.

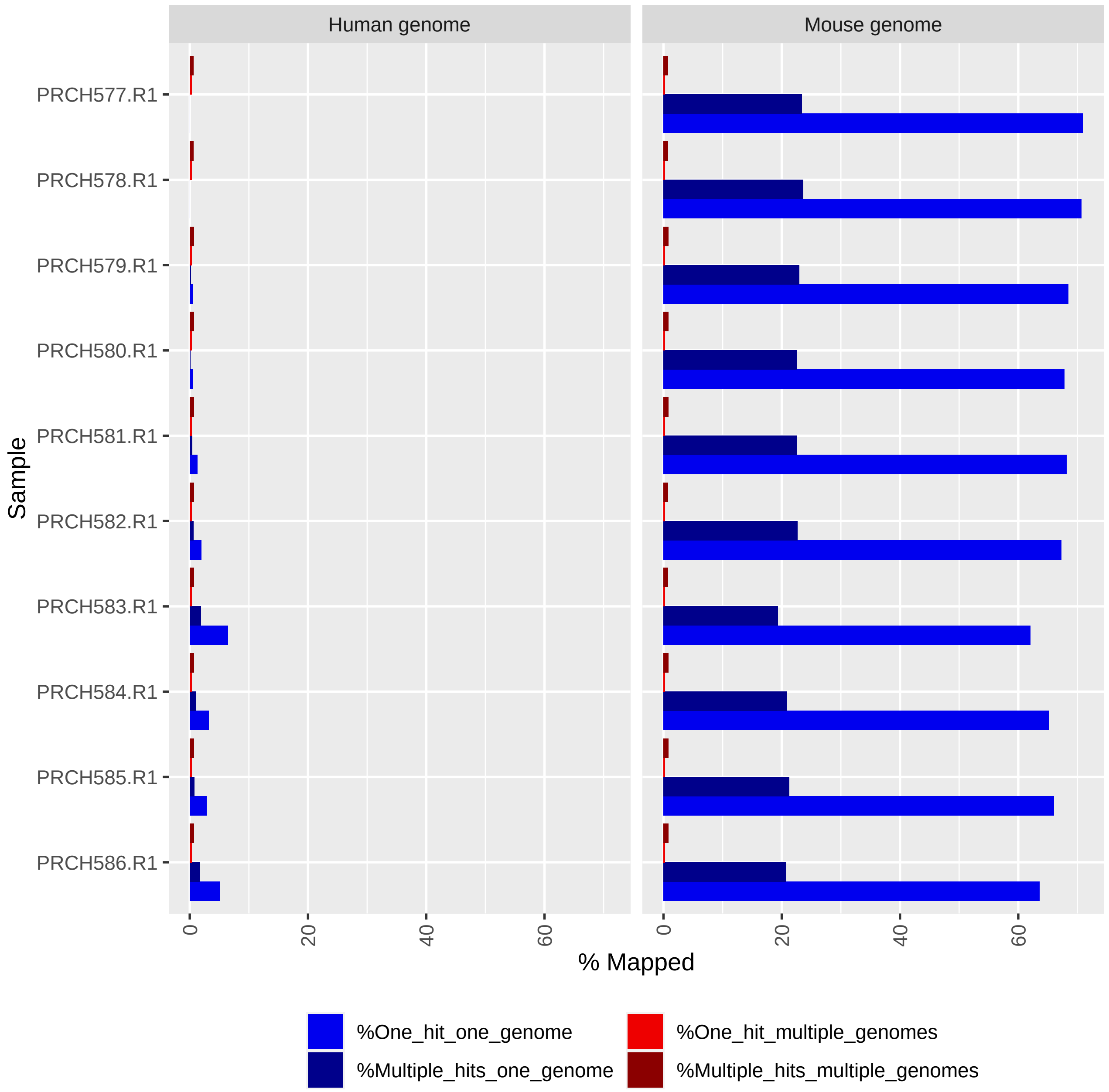


Figure 11: **Library screen for read 1.** This figure represents, for each sample, the percentage of reads aligned to the DNA sequence indicated on the top of the figure. The light blue and dark blue (light red and dark red) colors indicate reads which can be aligned to only (more than) one DNA sequence(s). The light blue and light red (dark blue and dark red) colors indicate reads that can be aligned to only (more than) one position onto the DNA sequence(s).

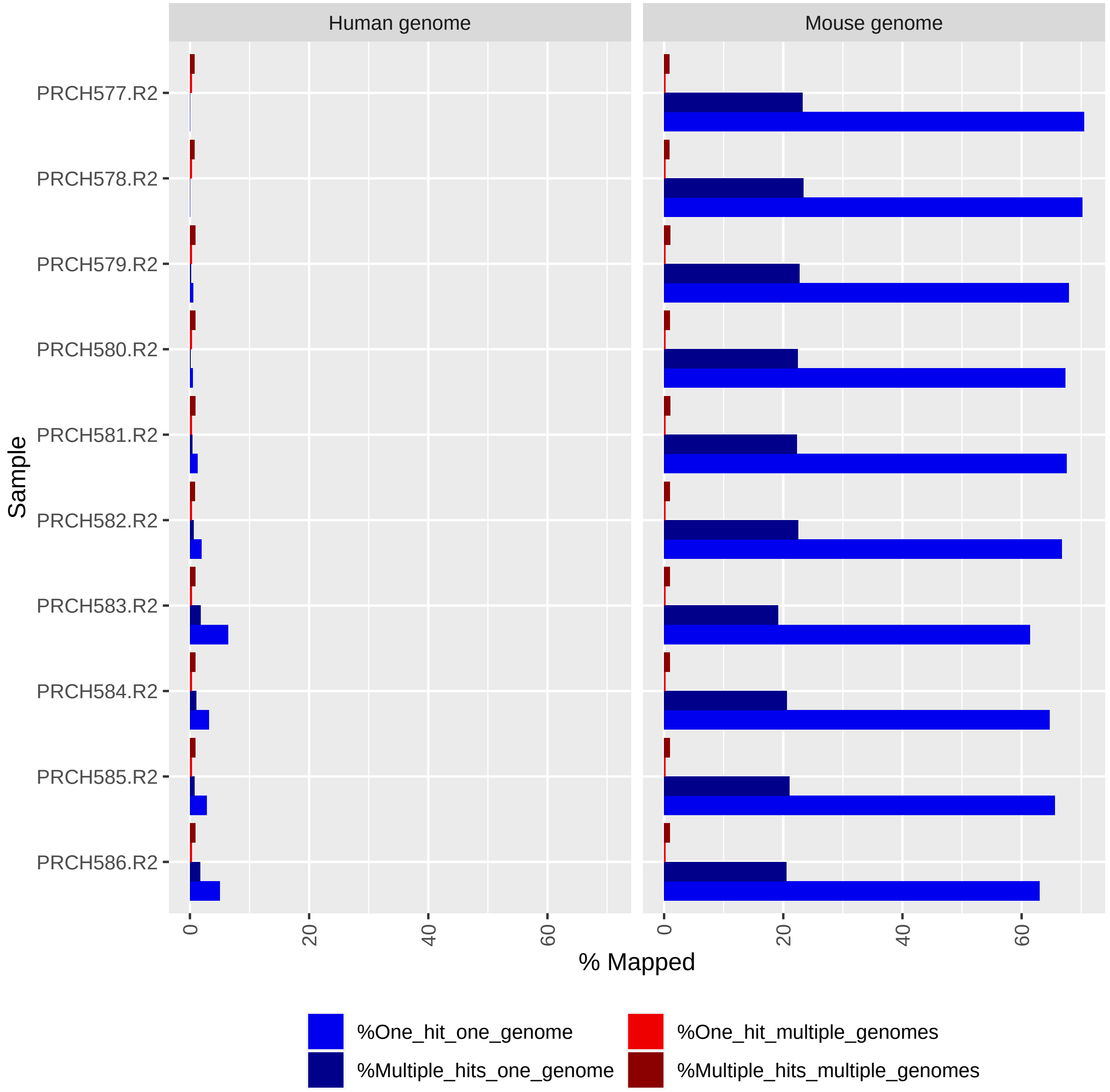


Figure 12: **Library screen for read 2.** This figure represents, for each sample, the percentage of reads aligned to the DNA sequence indicated on the top of the figure. The light blue and dark blue (light red and dark red) colors indicate reads which can be aligned to only (more than) one DNA sequence(s). The light blue and light red (dark blue and dark red) colors indicate reads that can be aligned to only (more than) one position onto the DNA sequence(s).

Table 3: **Size of available files.**

| File | Size |
|---|---|
| PRCH577.R2.fastq.gz | 1.74 GB |
| PRCH577.R1.fastq.gz | 1.73 GB |
| PRCH578.R2.fastq.gz | 1.77 GB |
| PRCH578.R1.fastq.gz | 1.76 GB |
| PRCH579.R2.fastq.gz | 1.22 GB |
| PRCH579.R1.fastq.gz | 1.21 GB |
| PRCH580.R2.fastq.gz | 1.73 GB |
| PRCH580.R1.fastq.gz | 1.72 GB |
| PRCH581.R2.fastq.gz | 1.40 GB |
| PRCH581.R1.fastq.gz | 1.38 GB |
| PRCH582.R2.fastq.gz | 1.45 GB |
| PRCH582.R1.fastq.gz | 1.45 GB |
| PRCH583.R2.fastq.gz | 1.55 GB |
| PRCH583.R1.fastq.gz | 1.53 GB |
| PRCH584.R2.fastq.gz | 1.21 GB |
| PRCH584.R1.fastq.gz | 1.20 GB |
| PRCH585.R2.fastq.gz | 2.37 GB |
| PRCH585.R1.fastq.gz | 2.35 GB |
| PRCH586.R2.fastq.gz | 1.48 GB |
| PRCH586.R1.fastq.gz | 1.47 GB |
| md5_240703.txt | 1.05 KB |
| Size of all files | 31.74 GB |

Table 4: **Format and content of available files.**

| File | Format | Content |
|---|---|---|
| *.fastq.gz | FASTQ, compressed with gzip | Reads and their qualities in Sanger format |
| md5_*.txt | Text file | MD5 for each file |

Table 3 lists all files that are available on the FTP server with their size and table 4 explains the format and the content of each type of file.

It is your responsibility to download, verify the integrity[3] and store these data. They will be required if you would like to publish results from these data. They will be deleted in 6 months, without any possibility for us to recover these data.

## 6 Publication

If you would like to publish these data, the following text can be used in the material and methods section: Library preparation was performed at the GenomEast platform at the Institute of Genetics and Molecular and Cellular Biology using Diagenode MicroPlex Library Preparation kit v3 Instruction Manual. ChIP samples were purified using SPRIselect beads (Beckman-Coulter, Villepinte, France) and quantified using the Qubit 4 fluorimeter (Thermo Fischer Scientific, Illkirch, France). ChIP-seq libraries were prepared from 10 ng of double-stranded purified DNA using the MicroPlex Library Preparation kit v3 (C05010001, Diagenode, Seraing, Belgium), according to manufacturer's instructions. In the first step, the DNA was repaired and yielded molecules with blunt ends. In the next step, stem-loop adaptors with blocked 5 prime ends were ligated to the 5 prime end of the genomic DNA, leaving a nick at the 3 prime end. The adaptors cannot ligate to each other and do not have single-strand tails, avoiding non-specific background. In the final step, the 3 prime ends of the genomic DNA were extended to complete library synthesis and Illumina

[3] To verify the integrity of your data after download, you can use the MD5 strings available in md5.txt file. If you need more information on MD5, please consult the following webpage : `http://genomeast.igbmc.fr/wiki/doku.php?id=help:md5`

compatible indexes were added through a PCR amplification (7 cycles). Amplified libraries were purified and size-selected using SPRIselect beads (Beckman Coulter) to remove unincorporated primers and other reagents.

Libraries were sequenced on an Illumina NextSeq 2000 sequencer as paired-end 50 base reads. Image analysis and base calling were performed using RTA version 2.7.7 and BCL Convert version 3.8.4.

If you would like to submit your data to Gene Expression Omnibus (GEO), please consult the following webpage : `https://www.ncbi.nlm.nih.gov/geo/info/seq.html`. A pre-filled GEO metadata spreadsheet is available on the FTP server.

If you could cite the platform in publication using these data, we will be able to include your publication in reports for our funding agencies. This is important for the visibility of the platform and for further getting funding. We suggest you the following statement: "Sequencing was performed by the GenomEast platform, a member of the 'France Genomique' consortium (ANR-10-INBS-0009)". We thank you in advance to tell us when such an article is published, so that we can inform our funding organisms.

# 7 Methods

Table 5: **Tools used for the analyses presented in this report.**

| Tool | Description | Version | Parameters |
| --- | --- | --- | --- |
| BCL Convert | To perform demultiplexing. | 3.8.4 | - |
| Bowtie | Used by FastQScreen to align reads on the genomes. | 1.3.1 | - |
| DPAPipe | Pipeline used to perform the primary analysis. | 3.1.0 | - |
| DPAPipeProject | Pipeline used to concatenate the data from different flowcells and to generate the report. | 2.4.0 | - |
| FastQC | To perform quality controls on the reads. | 0.11.5 | –threads : 1, –casava : True, –nogroup : True |
| FastQScreen | To screen a library of sequences in FASTQ format against a set of sequence databases. | 0.15.2 | –threads : 2, –subset : 10000000, –aligner : bowtie |
| R | To perform statistical analysis, graphics and to generate this report. | 4.1.1 | –vanilla : True |
| RTA | To extract intensities from images, perform base calling and assign a quality score to the base call. | 2.7.7 | - |

Table 5 provides the tools used in GenomEast DPAPipe (used to perform primary analyses described in the report) and their corresponding version. This pipeline was developed and is maintained by Damien Plassard, under the supervision of Céline Keime and Matthieu Jung.

# 8 Contributors

Wet lab operator: David RODRIGUEZ
Data processing and report operator: Bernard JOST

# References

[1] S. Andrew, "Fastqc." `http://www.bioinformatics.babraham.ac.uk/projects/fastqc/`, 2010.

[2] S. Wingett, "Fastqscreen." `http://www.bioinformatics.babraham.ac.uk/projects/fastq_screen/`, 2011.

[3] B. Langmead, "Ultrafast and memory-efficient alignment of short dna sequences to the human genome," *Genome Biology*, vol. 10, 2009.
