# Supplementary material for: IRX3 controls a SUMOylation-dependent differentiation switch in adipocyte precursor cells
Source: Nat Commun. 2025 Aug 6;16:7248. doi: 10.1038/s41467-025-62361-1 (PMC12328774; doi:10.1038/s41467-025-62361-1)
Supplement: Supplementary file 3 — Description of Additional Supplementary Files. [file 41467_2025_62361_MOESM3_ESM.pdf]

Description of Additional Supplementary Files

Bjune et al.,

IRX3 controls a SUMOylation-dependent differentiation switch in adipocyte precursor cells

File name: Supplementary Data 1
Description: Excel file related to IRX3 ChIP-seq in WAT.

File name: Supplementary Data 2
Description: HTML file showing IRX3 MEME motif discoveries under IRX3 ChIP-seq peaks.

File name: Supplementary Data 3
Description: HTML file showing TOMTOM matches to MEME discoveries for the IRX3 ChIP-seq.

File name: Supplementary Data 4
Description: Excel file summarizing TOMTOM results and their GO annotations for the IRX3 ChIP-seq.

File name: Supplementary Data 5
Description: Excel file showing ATAC-seq GO annotations for gWAT and ME3 cells.

File name: Supplementary Data 6
Description: Excel file showing data related to direct IRX3 target genes.

File name: Supplementary Data 7
Description: Excel file showing SUMO2/3 ChIP-seq counts and FCs in ME3 control and IRX3-KO cells.

File name: Supplementary Data 8
Description: Excel file showing GOs for differential SUMO2/3 binding in ME3 control and IRX3-KO cells.

File name: Supplementary Data 9
Description: HTML file showing STREME motif discoveries under SUMO2/3 ChIP-seq peaks.

File name: Supplementary Data 10
Description: Excel file summarizing TOMTOM results and their GO annotations for the SUMO2/3 ChIP-seq.

File name: Supplementary Data 11
Description: Excel file showing overlap and GO annotations for genes responding to ML-792 in 3T3-L1 cells and IRX3-KO in ME3 cells.

File name: Supplementary Data 12
Description: Excel file showing overlap between direct SUMO2/3 target genes in 3T3-L1 cells and IRX3-responsive genes in ME3 cells.

File name: Supplementary Data 13
Description: Excel file summarizing DEGs following ML-792 and IRX3-KO on different days of differentiation, all in ME3 cells. Overlap between sets of DEGs shown.

File name: Supplementary Data 14
Description: Excel file showing GOs for DEGs between IRX3-KO vs control ME3 cells.

File name: Supplementary Data 15
Description: Excel file showing GOs for DEGs between ML-792 vs DMSO treated ME3 cells.

File name: Supplementary Data 16
Description: Excel file showing GOs for inversely regulated genes between ML-792- and IRX3-KO-sensitive genes.

File name: Supplementary Data 17
Description: Excel file related to ATAC-seq in ME3 control and IRX3-KO cells. Differential peaks shown.

File name: Supplementary Data 18
Description: Excel file showing GO annotations for differential ATAC-seq peaks between IRX3-KO vs control ME3 cells.

File name: Supplementary Data 19
Description: Excel file showing overlap between RNA-seq and ATAC-seq data in IRX3-KO ME3 cells.
