# Supplementary material for: IRX3 controls a SUMOylation-dependent differentiation switch in adipocyte precursor cells
Source: Nat Commun. 2025 Aug 6;16:7248. doi: 10.1038/s41467-025-62361-1 (PMC12328774; doi:10.1038/s41467-025-62361-1)
Supplement: Supplementary file 23 — Reporting Summary [file 41467_2025_62361_MOESM23_ESM.pdf]

nature portfolio

Corresponding author(s): Pierre Chymkowitch, Gunnar Mellgren and Simon N. Dankel

Last updated by author(s): Jun 30, 2025

Reporting Summary

Nature Portfolio wishes to improve the reproducibility of the work that we publish. This form provides structure for consistency and transparency in reporting. For further information on Nature Portfolio policies, see our Editorial Policies and the Editorial Policy Checklist.

Statistics

For all statistical analyses, confirm that the following items are present in the figure legend, table legend, main text, or Methods section.

n/a	Confirmed	
☐	☒	The exact sample size (*n*) for each experimental group/condition, given as a discrete number and unit of measurement
☒	☐	A statement on whether measurements were taken from distinct samples or whether the same sample was measured repeatedly
☐	☒	The statistical test(s) used AND whether they are one- or two-sided<br>*Only common tests should be described solely by name; describe more complex techniques in the Methods section.*
☒	☐	A description of all covariates tested
☐	☒	A description of any assumptions or corrections, such as tests of normality and adjustment for multiple comparisons
☐	☒	A full description of the statistical parameters including central tendency (e.g. means) or other basic estimates (e.g. regression coefficient) AND variation (e.g. standard deviation) or associated estimates of uncertainty (e.g. confidence intervals)
☐	☒	For null hypothesis testing, the test statistic (e.g. *F*, *t*, *r*) with confidence intervals, effect sizes, degrees of freedom and *P* value noted<br>*Give P values as exact values whenever suitable.*
☒	☐	For Bayesian analysis, information on the choice of priors and Markov chain Monte Carlo settings
☒	☐	For hierarchical and complex designs, identification of the appropriate level for tests and full reporting of outcomes
☒	☐	Estimates of effect sizes (e.g. Cohen's *d*, Pearson's *r*), indicating how they were calculated

Our web collection on statistics for biologists contains articles on many of the points above.

Software and code

Policy information about availability of computer code

Data collection

Provide a description of all commercial, open source and custom code used to collect the data in this study, specifying the version used OR state that no software was used.

Data analysis

- IRX3 ChIP-seq reads were aligned to mouse genome (mm10) using Bowtie2 (Version 2.3.4.3)
- Aligned files were filtered using samtools (Version 1.7-2)
- ChIP-seq peak files were annotated using the R package ChIPseeker (Version 1.22.0)
- Log2 ChIP-over-input tracks for each alignment file were generated using deepTools bamCompare (Version 3.1.2)
- Bam files were submitted to Macs2 (Version 2.1.1) for peak calling
- ChIP-seq peaks were overlapped with peaks found in the ATAC-Seq data using the genomicRanges R package (Version 1.38.0)
- GOs analyzes were performed using Panther and Reactome pathway databases

SUMO2/3 ChIP-seq reads were mapped to mouse genome (mm10) using the ENCODE ChIP-seq pipeline (v1.6.1) that uses bowtie2 (v2.3.4.3) with default paramteters.
- samtools view (v1.9) was used to remove unmapped, low quality (MAQ <30) and duplicate reads with the following parameters "-F 1804 -q 30" and "-F 1804
- SUMO peak calling was performed with ssp v1.15.5 according to the ENCODE ChIP-seq pipeline v1.6.1
- Peaks were annotated relative to genomic features using Homer (v4.11)
- Functional enrichment analysis was performed on differential regions using ClusterProfiler (V4.10.1)
- Heatmaps were generated using Deeptools computeMatrix (v3.5.4) and plotHeatmap v3.5.4
- Mean profiles were generated using plotProfile (v3.5.4) and upset plots were made using ComplexHeatmap (v2.18.0)

- Known or de novo TF motifs were identified using HOMER findMotifsGenome.pl with default parameters.

- WT ME3 ATAC seq reads were aligned to the mm10 genome using Bowtie2 (Version 2.3.4.3) using the "very-sensitive" setting.

- ME3 control and IRX3-KO ATAC-seq reads were filtered using samtools Version 1.7-2 and picardTools Version 2.8.1.1
- Peak calling was performed via Macs2 (Version 2.1.1)
- Peaks were processed in Rsubread featureCounts (Version 1.6.0)
- Time-course analysis for each peak was performed via the R package maSigPro (Version 1.56.0). A regression fit for each peak was performed, and the p-value associated with the F-statistic of each model was calculated to uncover peaks which change dependent on experimental group. Peaks with an adjusted p-value less than 0.1 were then selected, and stepwise regression was performed to select significant variables for each gene. Peaks which were found to change over time were then submitted to hierarchical clustering and grouped into six clusters with differing time-dependencies and visualized via ggplot2 using a glm smoothing method with polynomial regression.
- differential ATAC-seq peaks were detected using Macs2 (Version 2.1.1) and sequencing reads within each individual peak were then counted using featureCounts and DESeq2.
- ME3 control and IRX3-KO RNA-seq reads were mapped to mouse genome (mm10) using Hisat2 and annotated using featureCounts. Normalization and differential expression analyses were performed using DESeq2.

- Other data analyzes were performed in GraphPad Prism (Version 10.3.0). Box plots show the median and interquartile range with whiskers indicating the minimum and maximum values, bar graphs show the mean values, with error bars representing SD. Statistically significant differences between group means were investigated using two-sided Student's unpaired t-test, one-way ANOVA or two-way ANOVA with Holm-Sidak correction for multiple testing as indicated. Data were tested for normality and homogeneity of variance, and transformed, if necessary, prior to statistical analysis to conform with the assumptions of the tests.

For manuscripts utilizing custom algorithms or software that are central to the research but not yet described in published literature, software must be made available to editors and reviewers. We strongly encourage code deposition in a community repository (e.g. GitHub). See the Nature Portfolio guidelines for submitting code & software for further information.

Data

Policy information about availability of data

All manuscripts must include a data availability statement. This statement should provide the following information, where applicable:

- Accession codes, unique identifiers, or web links for publicly available datasets
- A description of any restrictions on data availability
- For clinical datasets or third party data, please ensure that the statement adheres to our policy

Source data are provided with this paper. Sequencing data generated in this study are available at www.ebi.ac.uk/arrayexpress under accession codes
- E-MTAB-13524 [https://www.ebi.ac.uk/biostudies/ArrayExpress/studies/E-MTAB-13524?query=E-MTAB-13524%20] (ChIP-seq IRX3 in gWAT and iWAT)
- E-MTAB-13540 [https://www.ebi.ac.uk/biostudies/ArrayExpress/studies/E-MTAB-13540?query=E-MTAB-13540%20] (ATAC-seq in gWAT and iWAT)
- E-MTAB-13520 [https://www.ebi.ac.uk/biostudies/ArrayExpress/studies/E-MTAB-13520?query=E-MTAB-13520%20] (ATAC-seq in WT ME3 cells)
- E-MTAB-13525 [https://www.ebi.ac.uk/biostudies/ArrayExpress/studies/E-MTAB-13525?query=E-MTAB-13525%20] (ATAC-seq in control and IRX3-KO ME3 cells)
- E-MTAB-14723 [https://www.ebi.ac.uk/biostudies/ArrayExpress/studies/E-MTAB-14723?query=E-MTAB-14723%20%20%20] (RNA-seq on days -1, 1 and 9 in ME3 control and IRX3-KO treated with ML-792)

And at https://www.ncbi.nlm.nih.gov/geo/ under accession code
- GSE278972 [https://www.ncbi.nlm.nih.gov/geo/query/acc.cgi?acc=GSE278972] (SUMO2/3 ChIP-seq in ME3 control and IRX3-KO cells).

Sequencing data used in this study that was derived from Bjune et al., Metabolism 2020 (DOI: 10.1016/j.metabol.2019.154014) are available at www.ebi.ac.uk/arrayexpress under accession code
- E-MTAB-8209 [https://www.ebi.ac.uk/biostudies/ArrayExpress/studies/E-MTAB-8209?query=E-MTAB-8209%20] (RNA-seq in ME3 control and IRX3-KO on days 1 and 7).

Research involving human participants, their data, or biological material

Policy information about studies with human participants or human data. See also policy information about sex, gender (identity/presentation), and sexual orientation and race, ethnicity and racism.

Reporting on sex and gender	*Use the terms sex (biological attribute) and gender (shaped by social and cultural circumstances) carefully in order to avoid confusing both terms. Indicate if findings apply to only one sex or gender; describe whether sex and gender were considered in study design; whether sex and/or gender was determined based on self-reporting or assigned and methods used. Provide in the source data disaggregated sex and gender data, where this information has been collected, and if consent has been obtained for sharing of individual-level data; provide overall numbers in this Reporting Summary. Please state if this information has not been collected. Report sex- and gender-based analyses where performed, justify reasons for lack of sex- and gender-based analysis.*
Reporting on race, ethnicity, or other socially relevant groupings	*Please specify the socially constructed or socially relevant categorization variable(s) used in your manuscript and explain why they were used. Please note that such variables should not be used as proxies for other socially constructed/relevant variables (for example, race or ethnicity should not be used as a proxy for socioeconomic status). Provide clear definitions of the relevant terms used, how they were provided (by the participants/respondents, the researchers, or third parties), and the method(s) used to classify people into the different categories (e.g. self-report, census or*

	administrative data, social media data, etc.)<br>*Please provide details about how you controlled for confounding variables in your analyses.*
Population characteristics	*Describe the covariate-relevant population characteristics of the human research participants (e.g. age, genotypic information, past and current diagnosis and treatment categories). If you filled out the behavioural & social sciences study design questions and have nothing to add here, write "See above."*
Recruitment	*Describe how participants were recruited. Outline any potential self-selection bias or other biases that may be present and how these are likely to impact results.*
Ethics oversight	*Identify the organization(s) that approved the study protocol.*

Note that full information on the approval of the study protocol must also be provided in the manuscript.

Field-specific reporting

Please select the one below that is the best fit for your research. If you are not sure, read the appropriate sections before making your selection.

[x] Life sciences [ ] Behavioural & social sciences [ ] Ecological, evolutionary & environmental sciences

For a reference copy of the document with all sections, see nature.com/documents/nr-reporting-summary-flat.pdf

Life sciences study design

All studies must disclose on these points even when the disclosure is negative.

Sample size	For the mouse experiment, n = 12-24, in which adipose tissues from 6-12 animals were pooled and digested to obtain sufficient amount of cells for in vitro differentiation to allow for n = 2 samples for IRX3 ChIP-seq and n = 3 for ATAC-seq according to common practice. It should be noted that for gWAT, we were only able to make a library of satisfying quality from n = 1 sample.<br><br>For ATAC seq from ME3 cell lines, n = 2 was used for WT cells to comply with common practice, but for subsequent experiments we increased this number to n = 3 for control and IRX3-KO cells to ensure even higher reliability and reproducibility.<br><br>For other cell culture experiments, n = 3 replicate wells were used. Becaues each well contains 1e4 - >1e5 cells, each well already represents a large population. Thus, the variation between wells is relatively low and n = 3 is sufficient to obtain statistically meaningful data.
Data exclusions	Outliers were identified by the ROUT test (Q = 1%), but were excluded only when associated with an obvious technical error.
Replication	Most experiments (not omics experiments) were performed in 3-4 independent experiments (with 3-4 replicate wells). The data were highly reproducible.<br><br>IRX3 ChIP-seq experiments in WAT were validated by IRX3 ChIP-qPCR in ME3 cells.
Randomization	The animal study was not an intervention study (all animals received the same treatment), thus animals were not randomized. Cells cultures were treated in a non-random manner by adding vehicle to the first replicate wells, followed by the experimental treatment.<br><br>During library prep for sequencing, samples orders were reordered to minimize batch effects.
Blinding	Investigators were not blinded during data collection: it was not relevant in the animal study since all animals received the same treatment. In cell culture experiments, we did not consider the benefit of blinding to outweigh the associated costs.

Reporting for specific materials, systems and methods

We require information from authors about some types of materials, experimental systems and methods used in many studies. Here, indicate whether each material, system or method listed is relevant to your study. If you are not sure if a list item applies to your research, read the appropriate section before selecting a response.

Materials & experimental systems

n/a	Involved in the study	
[ ]	[x]	Antibodies
[ ]	[x]	Eukaryotic cell lines
[x]	[ ]	Palaeontology and archaeology
[ ]	[x]	Animals and other organisms
[x]	[ ]	Clinical data
[x]	[ ]	Dual use research of concern
[x]	[ ]	Plants

Methods

n/a	Involved in the study	
[ ]	[x]	ChIP-seq
[x]	[ ]	Flow cytometry
[x]	[ ]	MRI-based neuroimaging

Antibodies

Antibodies used

- anti-IRX3 (ab25793, lot GR198517-1, Abcam)
- anti-SUMO2/3 (ab3742, polyclonal, Abcam)
- anti-SUMO1 (ab32058, Y299, Abcam)
- anti-ACTB (ab6276, AC-15, Abcam)
- anti-VINCULIN (ab18058, SPM227, Abcam)
- anti-SENP1 (ab236094, polyclonal, Abcam)
- anti-SAE1 (ab185949, EPR15397(B), Abcam)
- anti-AM (AbFlex) (AB_2793779, Active Motif)
- anti-GAPDH (MAB374, 6C5, Millipore)
- anti-SENP5 (19529-1-AP, polyclonal, Proteintech)
- anti-UBA2 (8688S, D15C11, Cell signaling)
- anti-UBC9 (4786S, D26F2, Cell Signaling)
- anti-mouse (554002, polyclonal, BD Biosciences)
- anti-rabbit (31460, polyclonal, Invitrogen)

All primary antibodies were used at 1:1000 dilution except anti-SENP5, anti-SAE1 and anti-VINCULIN, which were used at a 1:2000 dilution. Secondary antibodies were used at 1:7,500 or 1:10,000 dilutions.

Validation

- anti-IRX3 (ab25703, lot GR198517-1, Abcam). Tested and working on WB in Mouse by Abcam, covered by their product promise. Tested for specificity by siRNA knockdown and overexpression by us, as described in the rebuttal letter.

- anti-SUMO2/3 (ab3742, polyclonal, Abcam). Tested and working on IHC-P, WB and ICC/IF in humans by Abcam, predicted by them to work in mouse. Validated to work on ChIP-seq in mouse (PMID: 35100417). Cited in 104 other articles.

- anti-SUMO1 (ab32058, Y299, Abcam). Tested and working in mice on IP, WB and IHC-P, expected to work in ChIP, ICC/IF and Flow by Abcam. Cited in 98 articles.

- anti-ACTB (ab6276, AC-15, Abcam). Tested and working in mice on WB, expected to work on ICC/IF by Abcam. Sited in more than 23,220 publications.

- anti-VINCULIN (ab18058, SPM227, Abcam). Abcam: reacts with mouse, suitable for WB, flow, ICC/IF, IHC-P. sited in 177 articles.

- anti-SENP1 (ab236094, polyclonal, Abcam). Tested and working in mice on WB, expected to work on IHC-P and ICC/IF by Abcam. Sited in 4 articles.

- anti-SAE1 (ab185949, EPR15397(B), Abcam). Tested and working in mice on WB, expected to work on IHC-P and ICC/IF by Abcam. Sited in 4 articles.

- anti-AM (AbFlex) (AB_2793779, Active Motif). Validated by Active Motif to work on ChIP, WB and ELISA (species-independent). Sited in 5 articles.

- anti-GAPDH (MAB374, 6C5, Millipore). Validated by manufacturer on WB and ICC/IF in mouse. Cited in 699 articles.

- anti-SENP5 (19529-1-AP, polyclonal, Proteintech). Validated by manufacturer on WB, IHC-P and IP in mouse. Cited in 9 articles.

- anti-UBA2 (8688S, D15C11, Cell signaling). Validated by manufacturer on WB and IP in mouse. Cited in 4 articles.

- anti-UBC9 (4786S, D26F2, Cell Signaling). Validated by manufacturer on WB and IP in mouse. Cited in 31 articles.

Eukaryotic cell lines

Policy information about cell lines and Sex and Gender in Research

Cell line source(s)	ME3 cells were derived from MEFS of RB -/- mice (PMID: 7739541). The sex of these mice is not described. Primary cells from iWAT and gWAT were derived from C57BL/6NJ (B6N) mice of both sexes.
Authentication	The ME3 cells were a gift from Karsten Kristiansen and have not been further authenticated in our lab, except for the ability of the cells to express Ucp1 mRNA and protein (beige-like properties).
Mycoplasma contamination	Cells were not tested for mycoplasma
Commonly misidentified lines (See ICLAC register)	*Name any commonly misidentified cell lines used in the study and provide a rationale for their use.*

Animals and other research organisms

Policy information about studies involving animals; ARRIVE guidelines recommended for reporting animal research, and Sex and Gender in Research

Laboratory animals	C57BL/6NJ (B6N) mice at the age of 6-10 weeks
Wild animals	*Provide details on animals observed in or captured in the field; report species and age where possible. Describe how animals were caught and transported and what happened to captive animals after the study (if killed, explain why and describe method; if released, say where and when) OR state that the study did not involve wild animals.*
Reporting on sex	Animals of both sexes were used for ATAC-seq data.<br>Female mice were used for generating the IRX3 ChIP-seq data (41-42 days old)
Field-collected samples	*For laboratory work with field-collected samples, describe all relevant parameters such as housing, maintenance, temperature, photoperiod and end-of-experiment protocol OR state that the study did not involve samples collected from the field.*
Ethics oversight	The MRC Harwell Animal Welfare and Ethical Review Board (AWERB) approved the animal experiment.

Note that full information on the approval of the study protocol must also be provided in the manuscript.

Plants

Seed stocks	*Report on the source of all seed stocks or other plant material used. If applicable, state the seed stock centre and catalogue number. If plant specimens were collected from the field, describe the collection location, date and sampling procedures.*
Novel plant genotypes	*Describe the methods by which all novel plant genotypes were produced. This includes those generated by transgenic approaches, gene editing, chemical/radiation-based mutagenesis and hybridization. For transgenic lines, describe the transformation method, the number of independent lines analyzed and the generation upon which experiments were performed. For gene-edited lines, describe the editor used, the endogenous sequence targeted for editing, the targeting guide RNA sequence (if applicable) and how the editor was applied.*
Authentication	*Describe any authentication procedures for each seed stock used or novel genotype generated. Describe any experiments used to assess the effect of a mutation and, where applicable, how potential secondary effects (e.g. second site T-DNA insertions, mosiacism, off-target gene editing) were examined.*

ChIP-seq

Data deposition

- [x] Confirm that both raw and final processed data have been deposited in a public database such as GEO.
- [x] Confirm that you have deposited or provided access to graph files (e.g. BED files) for the called peaks.

Data access links<br>*May remain private before publication.*	ChIP-seqdata generated in this study are available at<br><br>- www.ebi.ac.uk/arrayexpress under accession code E-MTAB-13524 [https://www.ebi.ac.uk/biostudies/ArrayExpress/studies/E-MTAB-13524?query=E-MTAB-13524%20] (ChIP-seq IRX3 in gWAT and iWAT)<br><br>- https://www.ncbi.nlm.nih.gov/geo/ under accession code GSE278972 [https://www.ncbi.nlm.nih.gov/geo/query/acc.cgi?acc=GSE278972] (SUMO2/3 ChIP-seq in ME3 control and IRX3-KO cells).
Files in database submission	E-MTAB-13524:<br>Irx3_ChIP-seq_gWAT_day_1_INPUT<br>Irx3_ChIP-seq_gWAT_day_1

Irx3_ChIP-seq_iWAT_Day_-1_INPUT
Irx3_ChIP-seq_iWAT_Day_-1_a
Irx3_ChIP-seq_iWAT_Day_-1_b
Irx3_ChIP-seq_iWAT_Day_1_INPUT
Irx3_ChIP-seq_iWAT_Day_1_a
Irx3_ChIP-seq_iWAT_Day_1_b
Irx3_ChIP-seq_processed.txt
Irx3_ChIP-seq_all-samples.bed

GSE278972:
GSM8559034 WT_Input_Day-1
GSM8559029 Irx3-KO_Input_Day1
GSM8559035 WT_SUMO2-3_Day-1_1
GSM8559036 WT_SUMO2-3_Day-1_2
GSM8559037 WT_SUMO2-3_Day1_1
GSM8559038 WT_SUMO2-3_Day1_2
GSM8559030 Irx3-KO_SUMO2-3_Day-1_1
GSM8559031 Irx3-KO_SUMO2-3_Day-1_2
GSM8559032 Irx3-KO_SUMO2-3_Day1_1
GSM8559033 Irx3-KO_SUMO2-3_Day1_2
GSE278972_RAW.tar

Sample 24

Genome browser session
(e.g. UCSC)

No longer applicable

Methodology

Replicates

IRX3 ChIP-seq Day -1 iWAT: 2 replicates (two independent experiments)
IRX3 ChIP-seq Day 1 iWAT: 2 replicates (two independent experiments)
IRX3 ChIP-seq Day 1 gWAT: 1 replicate

SUMO2/3 ChIP-seq WT ME3 cells on day -1: 2 replicates (three 10-cm dishes were pooled for each replicate)
SUMO2/3 ChIP-seq WT ME3 cells on day 1: 2 replicates (three 10-cm dishes were pooled for each replicate)
SUMO2/3 ChIP-seq IRX3-KO ME3 cells on day -1: 2 replicates (three 10-cm dishes were pooled for each replicate)
SUMO2/3 ChIP-seq IRX3-KO ME3 cells on day 1: 2 replicates (three 10-cm dishes were pooled for each replicate)

Sequencing depth

IRX3 ChIP-seq: ~30 million pair-end reads pr sample
SUMO2/3 ChIP-seq: 39-78 million paired-end reads pr sample (See separate Report file)

Antibodies

ab25703, lot GR198517-1, Abcam.

Peak calling parameters

Please refer to the methods section in the manuscript for detailed descriptions

Data quality

Please refer to the methods section in the manuscript for detailed descriptions and the separate Report file

Software

Please refer to the methods section in the manuscript for detailed descriptions
